# Both live and dead *Enterococci* activate *Caenorhabditis elegans* host defense via immune and stress pathways

**DOI:** 10.1080/21505594.2018.1438025

**Published:** 2018-03-19

**Authors:** Grace J. Yuen, Frederick M. Ausubel

**Affiliations:** a Department of Molecular Biology, Massachusetts General Hospital, Boston, Massachusetts, United States of America; b Program in Immunology, Harvard Medical School, Boston, Massachusetts, United States of America; c Department of Genetics, Harvard Medical School, Boston, Massachusetts, United States of America

**Keywords:** *Caenorhabditis elegans*, *Enterococcus faecalis*, *Enterococcus faecium*, host-pathogen interactions, innate immunity, invertebrate models of infection, pathogen-associated molecular patterns, stress response

## Abstract

The innate immune response of the nematode *Caenorhabditis elegans* has been extensively studied and a variety of Toll-independent immune response pathways have been identified. Surprisingly little, however, is known about how pathogens activate the *C. elegans* immune response. *Enterococcus faecalis* and *Enterococcus faecium* are closely related enterococcal species that exhibit significantly different levels of virulence in *C. elegans* infection models. Previous work has shown that activation of the *C. elegans* immune response by *Pseudomonas aeruginosa* involves *P. aeruginosa-*mediated host damage. Through ultrastructural imaging, we report that infection with either *E. faecalis* or *E. faecium* causes the worm intestine to become distended with proliferating bacteria in the absence of extensive morphological changes and apparent physical damage. Genetic analysis, whole-genome transcriptional profiling, and multiplexed gene expression analysis demonstrate that both enterococcal species, whether live or dead, induce a rapid and similar transcriptional defense response dependent upon previously described immune signaling pathways. The host response to *E. faecium* shows a stricter dependence upon stress response signaling pathways than the response to *E. faecalis*. Unexpectedly, we find that *E. faecium* is a *C. elegans* pathogen and that an active wild-type host defense response is required to keep an *E. faecium* infection at bay. These results provide new insights into the mechanisms underlying the *C. elegans* immune response to pathogen infection.

## Introduction

Innate immunity is an evolutionarily ancient defense system that provides the first line of defense against invading microbes and is found in all plant and metazoan life. Innate immunity in both plants and metazoans is mediated in part by pattern recognition receptors (PRRs) that recognize invariant molecular structures shared by pathogens, called microbe-associated molecular patterns (MAMPs). Upon activation, PRRs trigger downstream signaling, which ultimately culminates in the expression of immune effectors that inhibit the infection. While much is known about the activation of immune signaling pathways via MAMPs in plants, insects, and vertebrates, little is known about whether the nematode *Caenorhabditis elegans* is able to perceive MAMPs. It is possible that *C. elegans* is blind to MAMPs, as it is a natural bacteriovore, and thus must be somewhat tolerized to invariant microbial molecules such as lipopolysaccharides, peptidoglycans, and flagellin. In one relevant study, similar immune responses were activated when *C. elegans* was fed either live or heat-killed *Staphylococcus aureus*, suggesting that that *C. elegans* might be able to perceive MAMPs [1]. However, the single Toll-Like Receptor in *C. elegans* (TOL-1) does not appear to be significantly involved in innate immune signaling in *C. elegans*, at least in intestinal epithelial cells where the primary immune response occurs [2, 3]. Thus, these results suggested that the host response to *S. aureus* is activated by MAMPs in a manner independent of Toll signaling [1], although TOL-1-mediated signaling in chemosensory neurons can affect pathogen avoidance behavior [4].

In addition to MAMP recognition, host recognition of pathogen-elicited damage is also thought to be a general mechanism by which the host innate immune response is activated in both plants and animals [5], even though the mechanisms by which pathogens mediate direct or indirect damage to their hosts are still poorly understood. In the case of *C. elegans*, infection by live *Pseudomonas aeruginosa*, but not heat-killed cells, activates an immune response, with the magnitude of the host response to *P. aeruginosa* infection correlating with the degree of virulence of the infecting strain [5]. Moreover, *P. aeruginosa*-mediated blocking of host protein synthesis appears to be sufficient to activate immunity [6–8]. More generally, there is mounting evidence that disruption of cellular homeostasis by a variety of external toxins or defects in the function of particular essential genes can lead to immune activation in *C. elegans* [9–11].

With these results in mind, we investigated the mechanism by which *C. elegans* recognizes infection by two enterococcal species, *E. faecalis* and *E. faecium*, which have distinct infection-related phenotypes. Previously, our laboratory demonstrated that whereas *E. faecalis* and *E. faecium* both accumulate in and cause distention of the intestine of *C. elegans*, only *E. faecalis* is able to form a persistent infection and kill *C. elegans* [12]. These observations suggested that *C. elegans* may be tolerant to an *E. faecium* infection, if the accumulation of *E. faecium* in the intestine reflects a pathogenic process, as opposed, for example, to a mechanical or structural impediment in clearing *E. faecium* cells from the intestine. In any case, the mechanisms by which these two enterococcal strains disrupt host physiology, the nature of the host response to these pathogens, and the defense pathways required for resistance to *Enterococcus* have been elucidated only to a limited extent.

In this study, through ultrastructural imaging, we observed that infection with either *E. faecalis* or *E. faecium* causes intestinal distention in the absence of obvious damage, although infection with *E. faecalis* impairs the *C. elegans* defecation rhythm. Using gene expression profiling, we identified a large overlap in the *C. elegans* genes activated or repressed by *E. faecalis* or *E. faecium*, many of which are regulated by immune signaling pathways. We show that expression of these genes can also be elicited by heat-killed *Enterococcus*, but not by other heat-killed bacteria, demonstrating that *C. elegans* may perceive enterococcal-encoded MAMPs. We also demonstrate that stress pathways are required for the regulation of a specific subset of host effectors following infection with *E. faecium*, but not *E. faecalis*. Finally, we find that *E. faecium* is capable of infecting and killing immunocompromised *C. elegans* mutants. Since large numbers of *E. faecium* cells accumulate in the intestine of wild-type *C. elegans* animals, these results suggest that wild-type worms employ immune-response pathways to establish tolerance to an *E. faecium* infection. These findings shed new light on the mechanisms underlying pathogen sensing in *C. elegans*.

## Results

### Both *E. faecalis and E. faecium* distend the *C. elegans* intestine in the absence of extensive host damage, but only *E. faecalis* causes a lethal infection

Confirming previous work [12], we found that *E. faecalis* strain MMH594 causes a lethal infection in wild-type *C. elegans* strain N2 in an established agar-based assay [13], whereas the enterococcal species *E. faecium* strain E007 does not (S1 Fig). Importantly, the lethality of *E. faecalis* is not solely a consequence of *in utero* hatching of eggs (“bagging”). Although the LT_50_ of *C. elegans* treated with *cdc-25.1* RNAi to induce maternal sterility and subsequently fed *E. faecalis* is longer than wild-type worms fed *E. faecalis*, the LT_50_ of *cdc-25.1* RNAi-treated worms fed *E. faecalis* was almost half that of *cdc-25.1* RNAi-treated worms fed *E. faecium* (LT_50_ = 8 days and 18 days, respectively) (S1 Fig).

**Figure 1.**
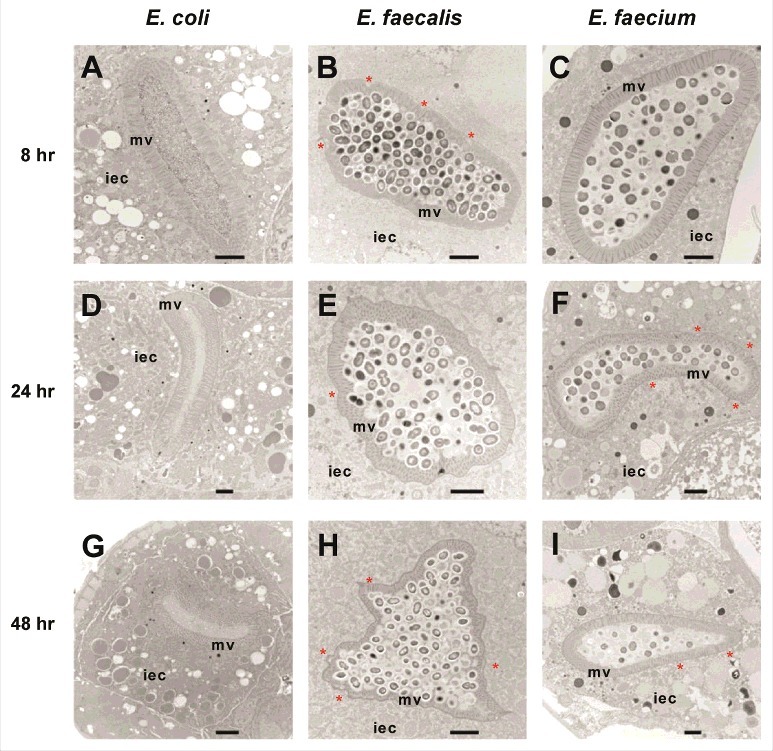
*Enterococci* proliferate in and cause distention of the *C. elegans* intestine, but do not invade intracellularly or lyse intestinal cells. Transmission electron micrographs of transverse midbody sections of N2 *C. elegans* feeding on *E. coli* OP50 (A, D, G), *E. faecalis* MMH594 (B, E, H), or *E. faecium* E007 (C, F, I) at 8 hours (A-C), 24 hours (D-F), or 48 hours (G-I) post infection. Representative micrographs are shown. The microvilli (mv) and cytoplasm of an intestinal epithelial cell (iec) are marked. Sites of dehiscence of the terminal web from the luminal membrane are marked with an asterisk. Scale bar, 2 µm.

We used transmission electron microscopy (TEM) to assess the ultrastructural cytopathology of *E. faecalis-* and *E. faecium*-infected worms. In control *E. coli*-fed *C. elegans* animals, nearly all bacterial cells in the intestinal lumen were macerated, and the intestinal microvilli appeared long, straight and anchored at their base into the terminal web (Figs. 1A, 1D, 1G). In contrast, *E. faecalis*- or *E. faecium* infected worms were found to have large numbers of bacterial cells packing and distending the intestine (Figs. 1B, 1E, 1H and Figs. 1C, 1F, 1I, respectively). Importantly, in both infections, many dividing bacterial cells were present in the intestine, as evidenced by the presence of septa, suggesting that they were actively proliferating. Interestingly, however, in the case of *E. faecium*, but not *E. faecalis*, many intestinal bacterial cells did not stain darkly, suggesting that their viability may have been negatively impacted by the host immune response. While the overall morphology of the intestinal cells in both *E. faecalis*- and *E. faecium* infected animals was not dramatically different from that observed in *E. coli*-fed worms, several features were clearly different in the *Enterococcus*-infected animals. First, some slight dehiscence of the terminal web from the luminal membrane was observed in the *E. faecium*-infected worms (Figs. 1C, 1F, 1I). Second, shortening of the intestinal microvilli could be seen in most *E. faecalis*-infected *C. elegans* (24 out of 32 sections) and some *E. faecium*-infected animals (7 out of 34 sections), which may be indicative of damage to the apical microvilli. Similar shortening of the microvilli was reported previously four days after infection with *E. faecalis* in a liquid-based killing assay [14]. Third, the basolateral surface of the intestinal cells of *E. faecalis* (Figs. 1E and 1H) and *E. faecium* (Figs. 1F and 1I) appeared undulatory in some TEM images (27 out of 32 *E. faecalis*-infected *C. elegans* sections; 10 out of 34 *E. faecium*-infected *C. elegans* sections), rather than exhibiting a smooth border as seen in worms fed *E. coli*. In contrast to live *E. faecalis* or live *E. faecium*, heat-killed enterococci did not accumulate in or distend the host intestine (data not shown). Furthermore, unlike infection by *E. faecalis* or *E. faecium*, previous TEM analysis showed that infection with *P. aeruginosa* or *S. aureus* caused severe intestinal damage [1]. *P. aeruginosa* infections were characterized by shortened microvilli, intracellular invasion from the intestine, and arrested autophagosomes, whereas *S. aureus* infections were characterized by severe effacement of the microvilli and lysis of intestinal epithelial cells [1]. All of the TEM images that we obtained are available upon request.

### *E. faecium* is a *C. elegans* pathogen but wild-type worms are at least partially tolerant to an *E. faecium* infection

**Figure 2.**
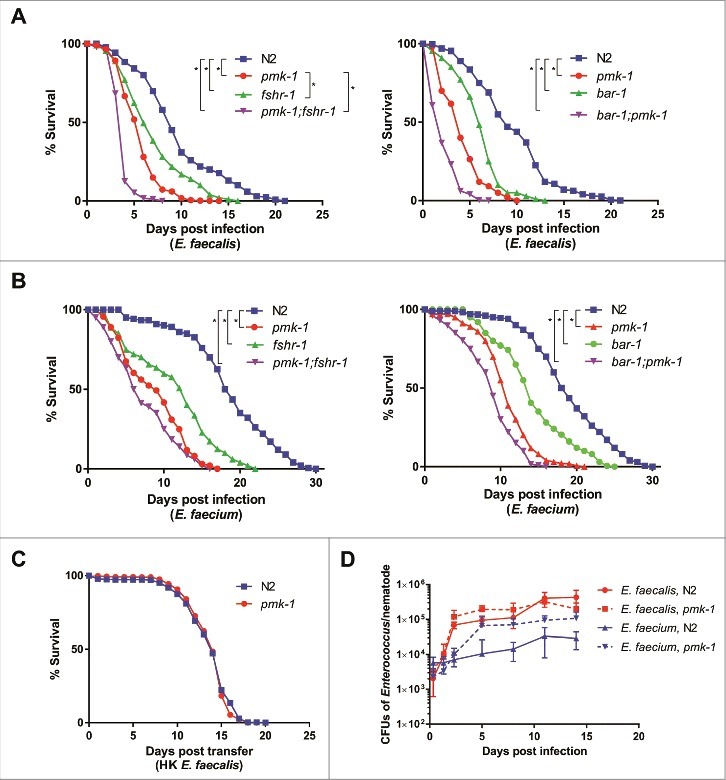
BAR-1, FSHR-1, and PMK-1 are required for defense against both *E. faecalis* and *E. faecium*. (A, B) Survival of wild-type N2, *pmk-1*(km25), *fshr-1*(ok778), and *pmk-1*(km25);*fshr-1*(ok778) *C. elegans* on *E. faecalis (left) and* wild-type N2, *pmk-1*(km25), *bar-1*(ga80), and *pmk-1*(km25);*bar-1*(ga80) animals (right) on *E. faecalis* (A) *and E. faecium* (B), sterilized with *cdc-25.1* RNAi. (C) Survival of N2 and *pmk-1*(km25) worms sterilized with *cdc-25.1* RNAi on heat-killed *E. faecalis*. Each graph shows the average of three plates for each strain, with each plate containing 30–40 worms. Results are representative of 3 independent assays. Statistical significance of differences between survival curves was calculated using Kaplan-Meier log rank analysis. Strains that showed a statistically significant difference from N2 are denoted with an asterisk and bracket. (D) Bacterial load of wild-type N2 and *pmk-1 C. elegans* after infection with *E. faecalis* or *E. faecium*. Time course of colony forming units in the intestines of wild-type N2 or *pmk-1* deficient *C. elegans*, sterilized with *cdc-25.1*, and infected with either *E. faecalis* or *E. faecium*. Error bars represent the standard deviation. Results are representative of 2 independent assays; each condition of each experiment was performed in triplicate or quadruplicate.

Previous work has shown that immune-deficient *C. elegans* exhibit enhanced susceptibility to *E. faecalis* [12]. We extended this work by assessing the survival of various characterized *C. elegans* immunity-related mutants feeding on either *E. faecalis* or *E. faecium*. In the case of *E. faecalis* infection, *pmk-1, fshr-1*, and *bar-1* mutants were more susceptible than wild-type worms (Fig. 2A). PMK-1 encodes a p38 MAPK that is a highly-conserved component of metazoan immune response pathways [15, 16]. FSHR-1 encodes a leucine-rich repeat containing G-protein coupled receptor, homologous to the human follicle stimulating hormone receptor, and is important in the *C. elegans* defense response against *P. aeruginosa* [17]; recently, it has also been shown to regulate the host response to oxidative stress [18]. BAR-1 encodes a *C. elegans* homolog of β-catenin critical for conferring resistance to *S. aureus* [19]. Interestingly, *pmk-1;fshr-1* and *pmk-1; bar-1* double mutants were more susceptible to *E. faecalis* than the single mutants, suggesting that BAR-1 and FSHR-1 may each function in pathways independent from PMK-1 to mediate *C. elegans* immunity against *E. faecalis* infection (Fig. 2A).

Unexpectedly, although *E. faecium* does not kill wild-type *C. elegans* (S1 Fig), we observed the same pattern of susceptibility among the *pmk-1, fshr-1*, and *bar-1* mutants after infection with *E. faecium* (Fig. 2B) as with *E. faecalis* (Fig. 2A). The *pmk-1* mutant was also susceptible to a different *E. faecium* strain (BM4105SS) (S2 Fig). Importantly, wild-type N2 and mutant *pmk-1* worms showed no difference in lifespan on heat-killed *E. faecalis* (Fig. 2C). Similarly, *pmk-1* and *fshr-1* showed no statistically significant difference in lifespan from wild-type N2 worms on the non-pathogenic food source heat-killed *E. coli*, although *bar-1* and both *pmk-1;fshr-1* and *pmk-1;bar-1* exhibited some decrease in survival (S3 Fig). These data demonstrate that an active immune response is required for defense against *E. faecium* and that *E. faecium* appears to be a *C. elegans* pathogen.

The observation that *E. faecium* accumulates to large numbers in the *C. elegans* intestine (Fig. 1) and that immune pathways are required for the maintenance of host viability (Fig. 2) suggested that *C. elegans* is at least partially tolerant of an *E. faecium* infection. While it is not possible to vary microbial load in the *C. elegans* system, as *C. elegans* are infected by feeding *ad libitum* on bacterial lawns and often ingest bacteria on the plates that they have previously egested, we measured the load of viable bacterial cells in the *C. elegans* intestine of worms infected with *E. faecalis* or *E. faecium* as determined by their ability to form colonies when the worms were disrupted (see Methods). We found that N2 worms accumulated up to 4.3 × 10^5^ *E. faecalis* CFUs in their intestines, whereas only a maximum of 3.1 × 10^4^ CFUs accumulated in case of *E. faecium* (Fig. 2D). This observation correlates with the TEM results in Fig. 1, suggesting that *E. faecium* may be more susceptible than *E. faecalis* to the *C. elegans* immune response. A deficiency in PMK-1, however, led to an increase in the number of *E. faecium* CFUs recovered per worm (up to 1.1 × 10^5^ cells/worm), but no significant increase in CFUs upon *E. faecalis* infection (up to 3.2 × 10^5^ cells/worm). Thus, bacterial CFUs per worm correlate with host killing in the case of *E. faecium*, but not *E. faecalis*, even though deficiency of PMK-1 causes *C. elegans* to prematurely succumb to either infection. The reason that *E. faecalis* does not accumulate to higher titers in a *pmk-1* mutant than in wild type worms may be that the intestine simply cannot accommodate any additional bacterial cells. Thus, it appears that wild-type *C. elegans* can tolerate a significant *E. faecium* infection, but that this level of tolerance requires PMK-1, and likely other components of the *C. elegans* immune response, which negatively impact *E. faecium* viability.

### *E. faecalis* infection perturbs *C. elegans* defecation

**Figure 3.**
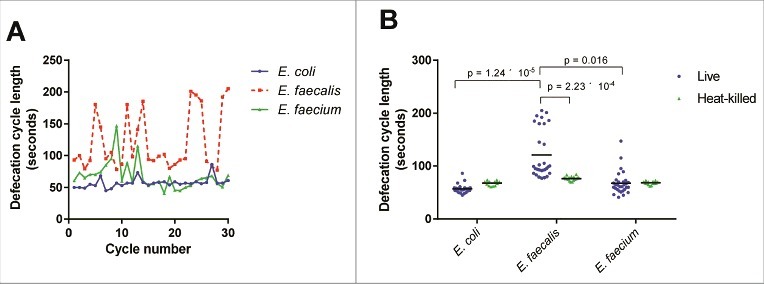
Live *E. faecalis* perturbs defecation cycle length in *C. elegans*. (A) Sequential defecation cycle lengths (time between consecutive contractions of the posterior body wall muscle) in wild-type *C. elegans* fed on *E. coli, E. faecalis*, or *E. faecium*. (B) Defecation cycle lengths in L4 worms fed *E. coli, E. faecalis*, or *E. faecium*, either live or heat-killed, for 24 hours. Mean defecation cycle lengths are indicated with horizontal bars. Results are representative of 3 independent assays. Relevant statistically significant differences are indicated with a bracket. Statistical significance was calculated using an unpaired t-test and the Holm-Sidak method for multiple comparison correction.

We hypothesized that the efficient colonization of the intestine by enterococci may be at least in part a consequence of a decreased defecation rate, as described for other Gram-positive bacterial infections [20]. Indeed, *E. faecalis*-infected *C. elegans* displayed a highly irregular and longer defecation rhythm than *E. coli*-fed worms (Fig. 3A). *E. faecium*-infected *C. elegans* exhibited a somewhat intermediate phenotype, and *C. elegans* fed on heat-killed bacteria, whether *E. coli, E. faecalis*, or *E. faecium*, had similar mean defecation cycle lengths (68.1, 76.5, and 68.6 seconds, respectively) (Fig. 3B). Although the underlying mechanism is not clear, these data suggest that the extent of disruption of the normal defecation rhythm correlates with worm killing, but that the relatively modest decrease in defection rate observed in the case of *E. faecium* is most likely not solely responsible for the accumulation of *E. faecium* in the *C. elegans* intestine.

### The *Enterococcus* infection gene signature

**Figure 4.**
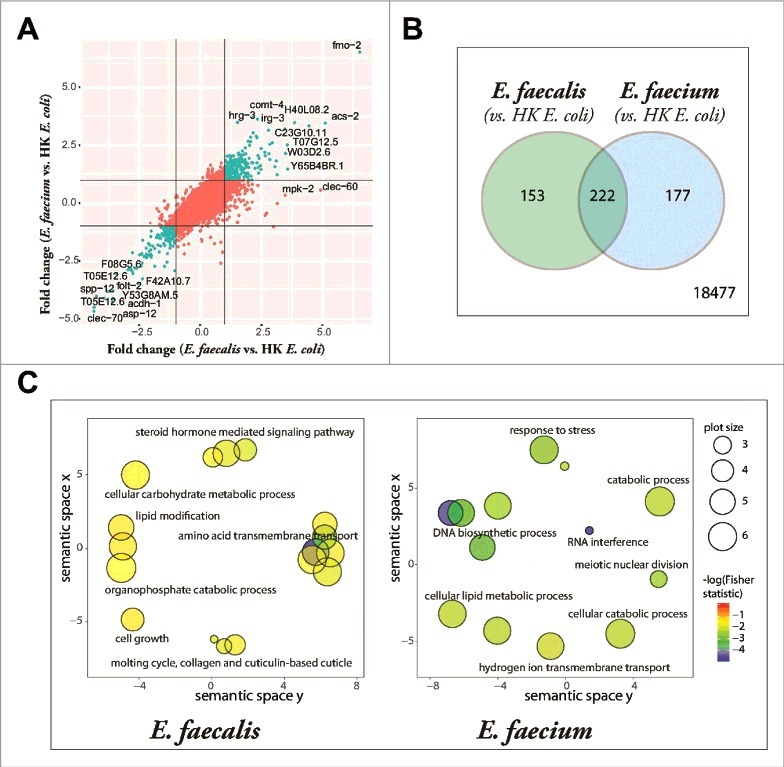
*Enterococcus* infection gene signature. (A) Fold-change by fold-change plot, depicting *E. faecalis*-infected *C. elegans* with *E. faecium*-infected *C. elegans* (relative to heat-killed *E. coli*-fed controls). Genes differentially expressed by both *E. faecalis* and *E. faecium* (relative to HK *E. coli*, |FC| > 2) are highlighted in aquamarine. 18,477 annotated Affymetrix probes were found to not be differentially expressed in either comparison. (B) The set of differentially expressed genes (|FC| > 2, BH-adjusted p-value < 0.05) in the *E. faecalis* infection signature (375) shared a significant overlap with genes differentially expressed in the *E. faecium* infection (399), p = 1.37 × 10^295^. (C) Functional classification summary of the differentially expressed genes that are exclusive to either *E. faecalis* (left) or *E. faecium* (right), relative to heat-killed *E. coli*. Similar functional categories of GO terms were clustered together in two-dimensional space using REViGO, a visualization tool that uses a clustering algorithm to represent GO data by plotting their semantic similarity. The bubble color indicates the Fisher test statistic while the bubble size is proportional to the frequency of GO terms in the GO annotation database. Colors corresponding to log10 Fisher test statistic are provided in the legend.)

We characterized the *C. elegans* host response to *Enterococcus* infection with whole-genome transcriptional profiling of *Enterococcus*-infected young adult *C. elegans* using Affymetrix GeneChip® technology. The transcriptional profile of *C. elegans* fed heat-killed *E. coli* was used as a control because live *E. coli* is pathogenic to *C. elegans* on BHI agar, the rich medium required for *E. faecalis* and *E. faecium* growth [12]. Using a fold-change cutoff of 2-fold (relative to heat-killed *E. coli*) and a Benjamini-Hochberg adjusted p-value of 0.05, we identified 375 differentially expressed genes (235 upregulated, 140 downregulated) in the *E. faecalis* infection compared to *E. coli* (S1 Table) and 399 differentially expressed genes (244 upregulated, 155 downregulated) in the *E. faecium* infection (S2 Table). Plotting the fold changes of the transcripts induced by *E. faecali*s against those induced by *E. faecium*, each relative to the heat-killed *E. coli* control, revealed a high degree of correlation between the two signatures, both for genes upregulated by both pathogens (upper right quadrant), as well as those downregulated by both pathogens (lower left quadrant) (Fig. 4A). The overlapping infection gene signature of *E. faecalis* and *E. faecium* contained 222 genes (p = 1.37 × 10^−295^, Fig. 4B and S3 Table) and was overrepresented by genes associated with amino acid metabolism, oxidation-reduction, lipid metabolism, and the innate immune response, as determined by gene ontology term analysis (S3 Table). Among the effector genes upregulated by infection with *Enterococcus* were three genes that had previously been demonstrated to mediate *C. elegans* resistance to pathogens: *sodh-1* (sorbitol dehydrogenase) and *cyp-37B1* (cytochrome P450), whose decreased expression caused enhanced susceptibility to killing by *S. aureus* [1], and *ilys-3*, an invertebrate lysozyme effector required in the pharynx and in the intestine to prevent the accumulation of bacterial cells in the gut lumen and to protect against *M. nematophilum* [21].

Although there were many similarities between the *E. faecalis* and *E. faecium* gene signatures, there were other facets of the gene signatures that differed between the two species. Using gene ontology term enrichment (Fig. 4C), we detected that the genes exclusively regulated by *E. faecalis* (and not *E. faecium*) revealed an enrichment in genes associated with amino acid transmembrane transport, lipid transport, steroid hormone mediated signaling, and a defense response (S4 Table), while transcripts exclusively regulated by *E. faecium* were enriched in genes associated with RNA interference, DNA biosynthesis and DNA repair, tRNA aminoacylation for protein translation, and the response to stress (S5 Table).

We hypothesized that genes regulated in response to infection by disparate pathogens may be responding to a shared facet of the infection process, such as signals indicative of host damage. Comparing the datasets of previously published transcriptional profiling with our datasets revealed a significant overlap between the genes regulated by both enterococci and three other pathogens (S4 Fig), *S. aureus* (S6 Table), *P. aeruginosa* (S7 Table), and even the fungal pathogen *C. albicans* (S8 Table).

When we directly compared the arrays from *C. elegans* infected with *E. faecalis* to those infected with *E. faecium*, we noted 18 Affymetrix probes for *C. elegans* transcripts that were more highly expressed in the *E. faecalis* infection relative to *E. faecium*, including the C-type lectins *clec-60* and *clec-82*, a number of hypothetical proteins (*e.g*., *H02F09.2, H02F09.3, C49C8.5*, and *Y54G2A.37*), and an F-box A protein (*fbxa-157*); only one transcript, the heme responsive gene *hrg-3*, was more highly expressed by *C. elegans* infected with *E. faecium* relative to *E. faecalis* (S9 Table). We reasoned that genes induced more highly in infection by *E. faecalis* than *E. faecium* may be responsive to the virulence of the infection. To address this issue, we compared the set of genes regulated by *E. faecalis* infection (relative to *E. faecium*) (S9 Table), to the set of genes differentially regulated by *Microbacterium nematophilum*, a *C. elegans*-specific Gram-positive bacterial pathogen, relative to an avirulent *M. nematophilum*, as this gene set may also include genes that are part of a “virulence signature”. This comparison yielded a small but statistically-significant overlap of three Affymetrix probes to two genes, *clec-60* and C49C8.5 (p = 2.27 × 10^−7^, S10 Table).

### Immune pathways are activated by and required for defense against E. faecalis and E. faecium

To validate the set of differentially-expressed genes identified by Affymetrix GeneChip^©^ technology, we designed a 72-gene “CodeSet” for the multiplexed NanoString nCounter® gene expression analysis system [22] that included genes activated or repressed exclusively by *E. faecalis* (13 genes), exclusively by *E. faecium* (7 genes), or both (47 genes) compared to heat-killed *E. coli*-fed controls, along with 5 “housekeeping” control genes. Even though the three different biological replicates tested in the NanoString experiment were a different set of biological replicates from those prepared for the Affymetrix microarray experiment, we observed an excellent concordance between the two experiments, for both *E. faecalis*- and *E. faecium*-induced genes (S5 Fig).

**Figure 5.**
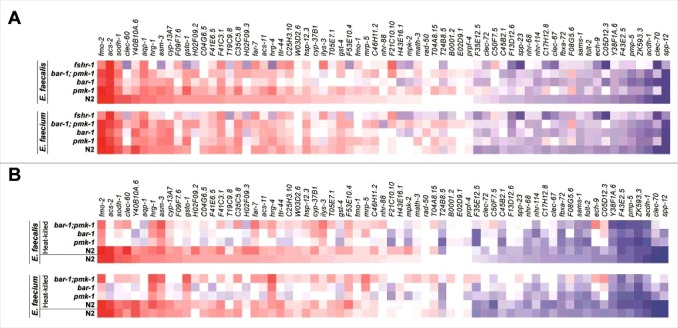
Induction of *Enterococcus*-activated genes through known *C. elegans* immune pathways and by heat-killed bacteria. (A) Heat-map of *Enterococcus*-activated genes in wild-type N2 worms or immune pathway mutant worms (*pmk-1*(km25), *bar-1*(ga80), *pmk-1*(km25)*;bar-1*(ga80), and *fshr-1*(ok778)), during infection with *E. faecalis* (top panel) or *E. faecium* (bottom panel). The heat-map reflects data from 2–3 biological replicates for each mutant analyzed. (b) Heat-map of *Enterococcus*-activated genes in wild-type N2 or immune pathway mutant worms, after 8 hours of exposure to heat-killed *E. faecalis* (top panel) or heat-killed *E. faecium* (bottom panel). For comparison, induction in N2 wild-type worms by live *E. faecalis* or *E. faecium* is shown in the bottom row of each panel. The heat-map reflects data from 2–3 biological replicates for each mutant analyzed. Genes are ordered by their degree of induction on *E. faecalis* in N2 worms, from red (most highly upregulated) to blue (most highly downregulated).

We used the NanoString CodeSet to investigate whether *Enterococcus*-induced genes are regulated by known immune-related pathways by profiling wild-type *C. elegans*, as well as mutants deficient in *pmk-1, bar-1*, or *fshr-1*, each infected with *E. faecalis* or *E. faecium* (Fig. 5A). These NanoString experiments led to four major conclusions. First, we found that the expression profiles of the 72 genes represented by the NanoString CodeSet in the different immune mutants were very similar for both *E. faecalis* and *E. faecium* (S6 Fig). This was at least partially expected given that the CodeSet was populated with some of the most highly upregulated and downregulated genes identified following *E. faecalis* or *E. faecium* infection, many of which overlapped between the two enterococcal species.

Second, the disruption of each of the three immune pathways hampered the induction of at least some of the *Enterococcus*-activated genes. Most strikingly, the *fshr-1* mutation abrogated expression of *clec-60, Y40B10A.6, hrg-1, asm-3, gsto-1, H02F09.2, hrg-4, W03D2.6*, and *cyp-37B1*, among a number of other genes, which was unexpected, because *fshr-1* was first identified as a regulator of a set of *P. aeruginosa* response genes, some of which are co-activated downstream of PMK-1 [17], and was not previously thought to play an important role in the response to Gram-positive bacterial pathogens.

Third, mutation of none of the *pmk-1, fshr-1*, or *bar-1* immune pathways abrogated the induction of all the *Enterococcus*-activated genes, though removal of any one of the pathways affected the induction of at least some of the *Enterococcus*-activated genes. Moreover, it appeared that wild-type expression of some genes (*e.g., clec-60, F09F7.6, T19C9.8, H02F09.2, H02F09.3*, and *T24B8.5*) depended at least in part on multiple pathways.

Fourth, some of the most highly upregulated genes in the *E. faecalis* infection appear to be negatively regulated by the *pmk-1, fshr-1*, and *bar-1* pathways (*e.g*., *clec-60, T19C9.8*, and *ilys-3*).

A separate NanoString experiment showed that the *pmk-1, fshr-1*, and *bar-1* pathways regulate *Enterococcus*-activated genes in the absence of pathogen attack (*i.e*., at steady state, when worms were only fed heat-killed *E. coli* OP50) (S7 Fig).

### Live and dead enterococci elicit similar gene expression profiles

Given the similarities between the *C. elegans* host response to *E. faecalis* and *E. faecium* infection, we hypothesized that the response might be driven by the perception of shared MAMPs between the two enterococcal species. To test this hypothesis, we used the NanoString CodeSet to compare the gene expression profile of N2 wild-type *C. elegans* fed heat-killed *E. faecalis* or *E. faecium* to that of N2 wild-type *C. elegans* fed live *E. faecalis* or *E. faecium*, respectively. The rationale for this experiment was that MAMPs are typically heat-stable components of microbial cell walls. Consistent with this hypothesis, we found the gene expression profiles elicited by live or dead *E. faecalis*, or live or dead *E. faecium*, to be extremely similar (Fig. 5B, bottom two rows of the upper and lower panels). Moreover, the induction of these genes by heat-killed *E. faecalis* or *E. faecium* was dependent on the immune regulators PMK-1 and BAR-1. Although the gene expression profiles of N2 worms fed live or heat-killed bacteria (*E. faecalis* or *E. faecium*) were highly correlated, the immune mutants clustered more closely based on whether they were exposed to heat-killed or live enterococci (S6 Fig), suggesting that there may be some conserved features of the response to heat-killed versus live Enterococci that are independent of the particular immune mutants.

Together, these gene expression profiling experiments show that *C. elegans* responds transcriptionally to live and dead *Enterococcus* similarly, and that full induction of these *Enterococcus*-activated genes are dependent upon known immune pathways. These results suggest that the perception of and response to *Enterococcus* is mediated by conserved heat-stable moieties, potentially including MAMPs synthesized by *E. faecalis* and *E. faecium*.

### *Enterococcus*-activated effectors are induced by both species, but are regulated by different pathways

**Figure 6.**
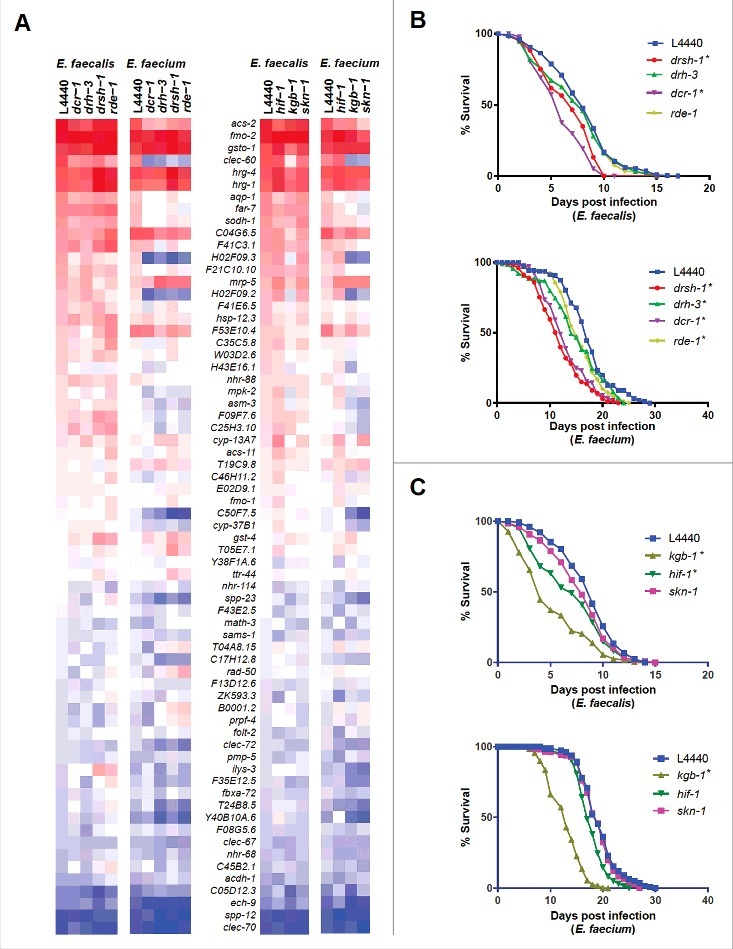
Stress response pathways are required for defense against *Enterococcus*. (a) Heat-map of the fold-changes of *Enterococcus*-activated genes in N2 worms treated with RNAi against *C. elegans* small RNA pathway components (*drsh-1, drh-3, dcr-1* or *rde-1*) (two panels on left) or stress response pathway components (*hif-1, kgb-1*, or *skn-1*) (two panels on right) or vector control (L4440) during infection with *E. faecalis* or *E. faecium* relative to heat-killed *E. coli* control. The heat-map reflects data from 2 biological replicates for each mutant analyzed. (b) Effect of *Enterococcus* infection on the survival of *fer-15*(b26);*fem-1*(hc17) *C. elegans* treated with L4440 vector control, *drsh-1, drh-3, dcr-1* or *rde-1* RNAi. (c) Effect of *Enterococcus* infection on the survival of *fer-15;fem-1 C. elegans* treated with L4440 vector control, *kgb-1, hif-1* or *skn-1* RNAi strains. For (b) and (c), each graph shows the average of three plates for each strain, with each plate containing 30–40 worms. results are representative of 3 independent assays and statistical significance of differences between survival curves was calculated using Kaplan-Meier log rank analysis. Relevant statistically significant survival curves (relative to control) are marked with asterisks.

From the gene ontology analysis (Fig 4C), we noted that genes related to RNA interference were enriched among the genes exclusive to the *E. faecium* infection signature. To test the importance of these genes in the host transcriptional response to *E. faecium*, we knocked down four RNAi-related genes that were differentially upregulated in the *E. faecium* infection signature: *dcr-1* (Dicer), *drh-3* (Dicer-related helicase-3), *drsh-1* (Drosha), and *rde-1* (one of the 27 *C. elegans* argonaute proteins). Knocking down these genes impaired the induction of a sizable subset of the *Enterococcus*-activated genes represented by the nCounter CodeSet (including *clec-60, aqp-1, far-7, H02F09.2, H02F09.3, H43E16.1, mpk-2, asm-3, F09F7.6*, and *C25H3.10*) following infection with *E. faecium*, but not *E. faecalis* (Fig. 6A, left two panels). Additionally, these small RNA pathway-related genes regulate the basal expression of the tested *Enterococcus*-activated genes when worms were feeding on *E. coli* OP50 (S8 Fig). To evaluate the role of the small RNA processing machinery in defense against *Enterococcus*, we knocked down the same four RNAi-related genes and then assayed for susceptibility of the worms to either *E. faecalis* or *E. faecium*. Worms deficient in either *dcr-1* or *drsh-1* exhibited enhanced susceptibility to *E. faecalis* or *E. faecium*; in contrast, *C. elegans* deficient in *drh-3* and *rde-1* were much more susceptible to *E. faecium* than *E. faecalis* (Fig. 6B). Importantly, knockdown of *drsh-1, drh-3*, and *rde-1* had no effect on lifespan on heat-killed *E. coli* and knockdown of *dcr-1* had only a marginal effect (S8 Fig). These results indicate that the small RNA processing pathway in *C. elegans* is required for defense against *Enterococcus*, but may be differentially required and regulated in defense against the two enterococcal species.

Because *dcr-1* mutants are compromised in a variety of stress response pathways [23], we also assessed the role of several stress-related regulatory genes in the induction of *Enterococcus*-activated genes. We found that knockdown of the JNK kinase KGB-1 [24] or the transcription SKN-1 [25], or to a lesser extent the transcription factor HIF-1 [26, 27] affected the induction of these genes in response to *E. faecium* but to a much lesser extent in *E. faecalis* (Fig. 6A, right two panels). Furthermore, the expression profiles of the RNAi-deficient worms following *E. faecium* infection were highly correlated to worms deficient in *skn-1* and *kgb-1* (S9 Fig). These stress-related genes also regulate the basal expression of *Enterococcus*-activated genes (S10 Fig), though not to the same degree as the previously examined immune pathways (PMK-1, BAR-1, *etc*.). Worms deficient in *kgb-1*, but not *skn-1* or *hif-1*, showed hypersensitivity to both enterococcal species (Fig. 6C). This was somewhat surprising because *skn-1* appears to play a larger role than *kgb-1* in the induction of the *Enterococcus-*activated genes following *E. faecium* infection. A caveat is that knockdown of *kgb-1* (but not *skn-1* or *hif-1)*, caused a partial decrease in lifespan on heat-killed *E. coli* (S10 Fig). Furthermore, the *skn-1* mutant has previously been shown to be hypersusceptible to *E. faecalis* infection [28, 29], and it is possible that knockdown by feeding RNAi may be generating only a weak “loss of function”. Taken together, these data suggest that canonical stress-response pathways play an important role in the *C. elegans* response to *E. faecium*.

### Nuclear hormone receptors contribute to the differential stress response of *E. faecalis* and *E. faecium*

**Figure 7.**
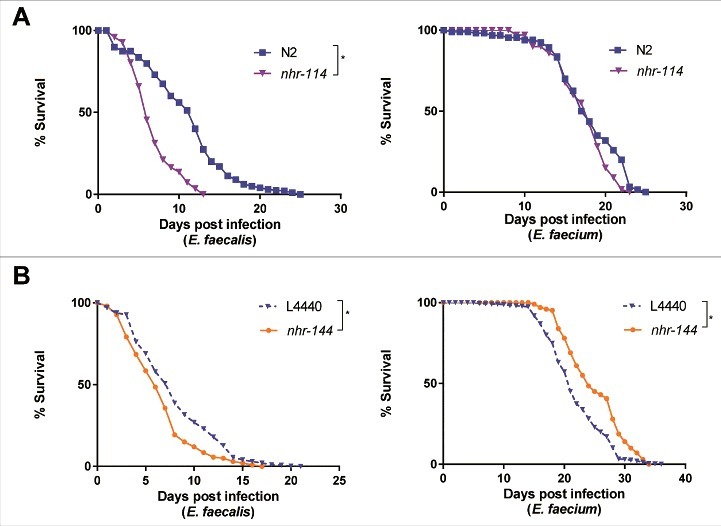
Nuclear hormone receptors are required for defense against *Enterococcus*. (a) Effect of *E. faecalis* and *E. faecium* infection on the survival of wild-type N2 and *nhr-114*(gk849). (b) Effect of *E. faecalis* and *E. faecium* infection on *fer-15*(b26);*fem-1*(hc17) *C. elegans* treated with L4440 vector control or *nhr-144* RNAi. Results are representative of 2 independent assays. Each graph shows the average of three plates for each strain, with each plate containing 30–40 worms. Relevant statistically significant survival curves (relative to control) are marked with asterisks and a bracket. Statistical significance of differences between survival curves was calculated using Kaplan-Meier log rank analysis.

Although stress-response pathways appear to be selectively required for the induction of immunity-related genes in response to *E. faecium* but not *E. faecalis* (Fig. 6A), only knockdown of *kgb-1* had a significant effect on the susceptibility to *E. faecalis* (Fig. 6C). We therefore reasoned that other pathways related to stress might explain the differential transcriptional response of *E. faecalis* and *E. faecium*. In this regard, we noted that steroid hormone mediated signaling pathway genes were enriched among the genes exclusively regulated by *E. faecalis* (S4 Table), many of which were nuclear hormone receptors (NHRs). NHRs comprise a family of transcription factors regulated by small lipophilic hormones and have been demonstrated to regulate gene expression in response to a variety of environmental signals [30]. To assess the role of a panel of the NHRs up- or downregulated by enterococcal infection (relative to heat-killed *E. coli* control) from the microarray data (S4 Table), we examined the survival of *C. elegans* in which these NHRs were mutated or knocked down using feeding RNAi (S11 Fig). Among the NHRs tested, we identified two, which when knocked down, exhibited opposite phenotypes following infection with *E. faecalis* or *E. faecium*: loss of function of *nhr-114* or knockdown of *nhr-144* led to increased susceptibility to *E. faecalis* infection (Fig. 7A and B, left), whereas loss of function of *nhr-114* had no effect on resistance to *E. faecium*, and knockdown of *nhr-144* led to modest but significant resistance to *E. faecium*, relative to worms fed an *E. coli* vector control (Fig. 7A and B, right). Importantly, loss of function of either NHR had no statistically significant effect on longevity on heat-killed *E. coli* (S12 Fig). These data suggest that nuclear hormone receptors and their downstream signaling partners may be playing an important role in immunity.

### Prior exposure of *C. elegans* to heat-killed *Enterococcus* confers *E. faecalis* resistance

**Figure 8.**
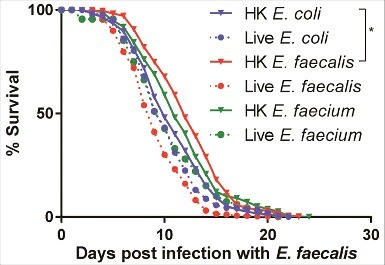
Pre-exposure to heat-killed *Enterococci* protects against subsequent infection with live *E. faecalis*. Survival of *fer-15*(b26)*;fem-1*(hc17) worms exposed to 8 hours of heat-killed bacteria (*E. coli, E. faecalis*, or *E. faecium*) or live bacteria (*E. faecalis* or *E. faecium*) and then transferred to live *E. faecalis*. Each graph shows the average of three plates for each condition, with each plate containing 30–40 worms. Results are representative of 2 independent assays. Statistically significant (*p*<0.05) survival curves that were increased in resistance (relative to HK *E. coli*) are marked with an asterisk and bracket. Statistical significance of differences between survival curves was calculated using Kaplan-Meier log rank analysis.

Because heat-killed *E. faecalis* or *E. faecium* are not pathogenic but induce a similar transcriptional response to live *E. faecalis* or *E. faecium*, we reasoned that pre-exposure of *C. elegans* to either heat-killed *E. faecalis* or *E. faecium* might render worms more resistant when subsequently challenged by a later *E. faecalis* infection, as hormeotic conditioning that protects from subsequent lethal infection has been described previously in worms [31–33]. We exposed *C. elegans* to heat-killed *E. coli, E. faecalis*, or *E. faecium*, or to live *E. faecalis* or *E. faecium* for eight hours, after which these “pre-exposed” worms were transferred to a lawn of live *E. faecalis* (Fig. 8). Indeed, conditioning with heat-killed *E. faecalis* afforded a statistically significant level of resistance to *E. faecalis* (34% increase in mean lifespan with heat-killed *E. faecalis* treatment vs. heat-killed *E. coli*, p = 0.04). Pre-treatment with heat-killed *E. faecium* also appeared to render *C. elegans* somewhat more resistant (32% increase in mean lifespan vs. heat-killed *E. coli*, p = 0.08). However, pre-treatment with either *B. subtilis* or *S. aureus*, alive or heat-killed, conferred no protection (S13 Fig). Furthermore, the extension in lifespan by pre-treatment with heat-killed *E. faecalis* was not conferred transgenerationally (S13 Fig). Although these data suggest that heat-killed *E. faecalis* and *E. faecium* elicit a potentially protective, pathogen-specific defense response that renders *C. elegans* modestly more resistant to subsequent infection with live *E. faecalis*, it is not clear whether this preconditioning induces a generalized protective stress response or a specific immune-driven response.

## Discussion

Previously published work from our laboratory showed that when *C. elegans* feed exclusively on either *E. faecalis* or *E. faecium*, large numbers of bacterial cells accumulate in the *C. elegans* intestine, which becomes grossly distended. However, only *E. faecalis* kills the worms, suggesting that *C. elegans* can normally tolerate a significant *E. faecium* intestinal infection and that distention of the intestinal lumen and packing with bacteria per se does not necessarily impact worm longevity. Here we extended these initial observations by transmission electron microscopic ultrastructural imaging, which showed that both *E. faecalis* and *E. faecium* pack the intestinal lumen. However, whereas the majority of *E. faecalis* cells appear to be viable since they stain darkly in the TEM images, a significant fraction of the *E. faecium* cells appear to be nonviable. This correlates with the observation that there are about 5 times as many *E. faecalis* cells that accumulate in individual worms and that can form colonies compared to *E. faecium* cells. Compared to wild-type worms, *pmk-1* immuno-deficient worms accumulate several fold more viable *E. faecium* cells, which correlates with the observation that the *pmk-1* mutant is killed by *E. faecium* whereas wild-type worms are tolerant to the infection. On the other hand, although the *pmk-1* mutant is also more susceptible to killing by *E. faecalis* than wild-type worms, the bacterial load is about the same. The simplest explanation of these data is that *C. elegans* tolerates an *E. faecium* load of ∼3 × 10^4^ cells/worm in a *pmk-1*-dependent manner without impairing worm longevity. In *pmk-1* mutant worms, *E. faecium* accumulates to about a 5-fold higher level than in wild-type worms, which negatively impacts worm longevity. The higher bacterial load in an *E. faecalis* infection than in an *E. faecium* infection correlates with a more severe decrease in longevity. The reason that *E. faecalis* does not accumulate to higher titers in a *pmk-1* mutant than in wild type may be that the intestine simply cannot accommodate any additional bacterial cells.

We also show by transmission electron microscopic ultrastructural imaging that intestinal distention, even late in infection, appears to occur in the absence of any extensive host damage, even though *E. faecalis* kills the worms. In contrast, previous ultrastructural studies have shown that *C. elegans* lethality caused by *P. aeruginosa* and *Staphylococcus aureus* is accompanied by severe host cellular damage [1]. Despite the fact that neither *E. faecalis* nor *E. faecium* caused any obvious host damage, genome-wide transcriptional profiling showed that both enterococcal species elicited a relatively robust immune response and that 45% of the *C. elegans* genes differentially upregulated by *E. faecalis* infection relative to the heat-killed *E. coli* control were also differentially upregulated by *E. faecium*.

Testing a subset of 67 genes differentially regulated by *E. faecalis, E. faecium* or both using nanoString analysis showed that heat-killed *E. faecalis* and heat-killed *E. faecium* elicited most of the same genes as live *E. faecalis* (Fig. 5B). These experiments suggest that the primary immune response elicited by *E. faecalis* and *E. faecium* is not activated by host damage but rather by heat-resistant pattern recognition molecules, although it is formally possible that heat-killed Enterococci may induce a DAMP-triggered response in the *C. elegans* intestine. Even though *C. elegans* – pathogen interactions have been studied now for almost 20 years, it is not known what bacterial MAMPs, if any, are recognized by *C. elegans*, or what, if any, PRRs exist in the *C. elegans* genome to detect pathogens. Previous studies using *Candida albicans* or *S. aureus* showed that the heat-killed pathogen induced many of the same *C. elegans* genes as a live infection. In contrast, in the case of *P. aeruginosa* live bacterial cells are required to elicit a *C. elegans* immune response that is proportional to the killing ability of the *P. aeruginosa* strain [1,6). Further studies showed that host cellular damage such as that caused by the inhibition of protein synthesis by *P. aeruginosa* exotoxin A was sufficient to elicit a robust immune response [6,7,11].

Consistent with the conclusion that heat-killed *E. faecalis* elicits a similar transcriptional response as live *E. faecalis*, preconditioning worms with heat-killed *E. faecalis* for just 8 hours was sufficient to extend host survival by 1.5 days following subsequent *E. faecalis* infection. This is a relatively modest shift in lifespan, but in the wild may afford the worms an evolutionary advantage to escape, survive, and lay more eggs. Hormeotic conditioning in *C. elegans* has been described previously in response to exposure to either pathogenic or avirulent strains of *E. coli –* which induce slightly different, although somewhat overlapping, protective immune responses – prior to infection with entero-pathogenic *E. coli* (EPEC) [32]. Other studies have shown that pre-exposure of *C. elegans* to the probiotic bacterium *Lactobacillus acidophilus* enhanced Gram-positive, but not Gram-negative, immune responses in the host [33]. Additionally, pre-treatment of *C. elegans* with *E. faecium*, but not *E. coli* or *B. subtilis*, appears to promote pathogen tolerance through secreted *E. faecium* peptidoglycan hydrolase SagA in a *tol-1* dependent manner [35]. Though *E. faecium* does not appear to promote pathogen tolerance to *E. faecalis* in our studies, this may be because the TOL-1 immune pathway is more important in the *S. typhimurium* defense response than in *E. faecalis*.

Importantly, even though *E. faecium* does not kill wild-type worms, mutating previously described immune or stress response signaling pathways allows *E. faecium* to kill the worms, demonstrating that *E. faecium* is a weak *C. elegans* pathogen. In other words, the activation of canonical immune and stress response pathways in wild type worms make them tolerant to an *E. faecium* infection.

Interestingly, we observed that the maximal lifespan of *C. elegans* fed live *E. faecium* was considerably longer that the lifespan of worms fed heat-killed *E. faecalis* (30 vs. 20 days) (compare Fig. 2B and Fig. 2C). One potential explanation is that *E. faecalis* makes a heat stable toxin that contributes to worm killing, whereas *E. faecium* does not. If *E. faecalis* makes such a toxin, its ability to kill would have to be PMK-1-independent, since wild-type and *pmk-1* mutant worms have the same lifespans on heat-killed *E. faecalis* (20 days) (Fig. 2C). Moreover, the presumptive toxin could not be primarily responsible for the bulk of the immune gene induction shared between the *E. faecalis* and *E. faecium* infection gene signatures, since *E. faecium* presumably does not make the toxin. It is also possible that such a toxin might be at least partially responsible for the priming effect observed with *E. faecalis*. Heat-stable toxins have previously been explored in *P. aeruginosa* infection of *C. elegans* [36–38], and have been postulated as an explanation for the observation that live and heat-killed *S. aureus* both elicited the expression of a panel of immune response genes [1]. An alternative explanation for the longer lifespan of live *E. faecium* compared to heat-killed *E. faecalis* is that *E. faecium* induces a particularly effective immune response that abrogates killing.

NanoString multiplexed gene expression analysis examining the expression of *E. faecalis* and *E. faecium* activated genes showed that the previously-described PMK-1, FSHR-1, and BAR-1 immune pathways contribute to host defense (Fig. 5) and that these immune and stress response pathways appear to activate many common genes (Figs. 5 and 6). In addition, genetic epistasis analysis showed that the PMK-1 and FSHR-1 and the PMK-1 and BAR-1 pathways appear to act independently of each other (Fig. 2). These data are consistent with previous studies showing that *C. elegans* has several parallel immune pathways that converge upon a set of shared, but nevertheless distinct set of putative immune effectors, presumably allowing *C. elegans* to integrate and fine-tune the defense response to various combination of MAMPs, DAMPs, and other elicitors. The activation of multiple pathways may also act as a safeguard against pathogens that attempt to subvert host defense by targeting an upstream signaling molecule.

One somewhat unexpected finding was that the FSHR-1 signaling pathway is activated by both *E. faecalis* and *E. faecium*, and that FSHR-1 is required for wild-type level of resistance to both pathogens. Another surprising finding was that the infection with *E. faecium* but not *E. faecalis* elicited a change in a variety of immune and stress response genes that appear to be dependent on SKN-1, KGB-1, and small RNA processing. One possible explanation is that in the case of an *E. faecium* infection, FSHR-1 activates a variety of stress and immune genes through the coordinated activity of the small RNA processing pathway, KGB-1, and SKN-1, whereas in *E. faecalis*, FSHR-1 activates these downstream putative effectors independently of the stress pathways.

Taken together, our study sheds new light on how *Enterococcus* infection disrupts host physiology, the differential response of *C. elegans* to *E. faecalis* and *E. faecium*, as well as potential ways in which *C. elegans* recognizes and responds to bacterial infection.

## Materials and methods

### Strains and growth conditions

All *C. elegans* strains were maintained on nematode growth media (NGM) and fed *E. coli* strain OP50, as previously described. The *C. elegans* strains used in this study are wild-type N2 Bristol, *pmk-1*(km25), *fshr-1* (ok778), *pmk-1*(km25); *fshr-1*(ok778), *bar-1*(ga80), *bar-1*(ga80);*pmk-1*(km25), *nhr-68* (gk708), *nhr-101* (gk586), and *nhr-114* (gk849). The *bar-1*(ga80);*pmk-1*(km25) mutant was a gift of Javier Irazoqui and the *pmk-1*(km25); *fshr-1*(ok778) was generated in our lab [17]; all others were obtained from the *Caenorhabditis* Genetics Center. Unless otherwise stated, the *E. coli, E. faecalis, E. faecium*, and *B. subtilis* strains used in this study correspond to strains OP50 [39], MMH594 [40], E007 [12], and PY79 *sigF*::kan (gift of Richard Losick), respectively.

### Nematode killing assays

For all enterococcal killing assays, starter cultures of *E. faecalis* or *E. faecium* strains were prepared from single colonies inoculated into 5 ml of BHI broth and were incubated for 6–8 hours with shaking at 37°C. Afterward, 10 μL of log phase cultures were spread onto 35 mm brain-heart infusion (BHI) agar plates containing 10 μg/ml kanamycin, and incubated at 37°C overnight (16–20 hours) [13]. Before use, the plates were allowed to equilibrate to room temperature. Approximately 40–50 late L4-staged *C. elegans* worms were then transferred from a lawn of *E. coli* OP50 on NGM medium to BHI medium-grown *Enterococcus*, taking care to transfer as little *E. coli* as possible from the maintenance plates to the killing plates. Nematodes were placed outside of the lawn on the bare agar. The plates were then incubated at 25°C, and every 24 hours, worms were examined for viability using a dissecting microscope. Worms that did not respond to a gentle touch with a platinum wire pick to the head, body, and tail were scored as dead. As *E. faecium*-infected-*C. elegans* do not move much when they are near death, even when prodded, special attention had to be paid to head movement and pharyngeal pumping to determine whether the nematodes were alive. Worms that did not move were scored as dead, counted, and picked off the killing plate; worms that died from crawling off the agar were also picked off the plate, but were censored from the assay. Worms that were found to be still moving were scored as alive and were also counted.

Each experimental condition was tested in triplicate. Kaplan-Meier log rank analysis was performed to determine the statistical significance of the difference in survival curves using OASIS [41], an online, publicly available tool that provides Kaplan-Meier estimates and mean/median survival time by based on censored survival data. Bonferroni-corrected p-values ≤ 0.05 were considered statistically significant.

### RNAi feeding experiments

RNAi constructs were obtained from the publicly available Ahringer RNAi library [41]. All clones were verified by sequencing. For RNAi experiments, starter cultures of RNAi expressing *E. coli* HT115 bacterial clones were grown overnight in LB (25 ug/ml carbenicillin and 10 ug/ml tetracycline) at 37°C, followed by further growth for 4–6 hours in a larger volume of LB (carbenicillin) at 37°C. NGM plates containing 5 mM IPTG and 100 μg/ml carbenicillin were then seeded with the double-stranded RNAi-expressing HT115 bacteria [42]. The dsRNA within the bacteria was induced over two days at room temperature, after which L1 worms, synchronized by hypochlorite treatment and L1 arrest [43], were added to the plates. Worms were fed through the L4 stage with dsRNA-expressing bacteria to target genes of interest.

### Defecation assays

N2 or *pmk-1* worms were grown to the L4 stage and were then picked to *Enterococcus* or *E. coli*-seeded plates and incubated at 25°C for 8 or 24 hours. For scoring, worms were then moved to room temperature, allowed to acclimate for 30–60 minutes, and their defecation phenotype was scored by assessing the time between expulsions (which are preceded by posterior and anterior body wall muscle contraction, and the contraction of enteric muscles in a normally regular pattern) [44]. Defecation cycles were also followed in individual L4 worms for 30 consecutive cycles for some experiments. In general, more than 20 worms were scored and experiments were performed in duplicate. The significance of differences in values between conditions was determined using unpaired two-tailed Student t tests, with unequal variance.

### RNA isolation

*C. elegans* N2 wild-type animals were synchronized by hypochlorite treatment and L1 arrested. Arrested L1 worms were allowed to grow on NGM media seeded with OP50 and grown at 20°C until they reached the young adult stage. Young adults were then washed three times in M9W buffer and transferred to BHI (10 µg/ml) plates seeded with heat-killed *E. coli* OP50, live *B. subtilis* PY79, live *E. faecalis* MMH594, or live *E. faecium* E007. Worms were treated as described for the killing assays, with the exception that approximately ∼2,000 worms were plated onto each 10-cm assay plate. After 8 hours at 25°C, the treated worms were washed three times in M9W, resuspended in TRI Reagent (Molecular Research Center, Cincinnati, OH), per the manufacturer's instructions, and frozen at −80°C. Once thawed, total RNA was prepared and then further purified through an RNeasy column (Qiagen). Three independent replicates of each treatment were isolated.

For the multiplexed gene expression profiling studies using NanoString nCounter, the same protocol was followed, except altering the *C. elegans* strain (genotype) or the type of bacteria, depending on the experiment. Two or three independent replicates of each treatment were carried out.

For feeding of *C. elegans* with heat-killed bacteria, bacteria were pelleted by centrifugation, washed thrice in a large excess of M9W, concentrated 25x and incubated at 95°C for 90–120 minutes. The heat-killed bacterial suspension was equilibrated to room temperature before it was added to BHI agar plates and allowed to dry under a sterile hood with heat. Once dry, these plates were allowed to once again equilibrate to room temperature before worms were added to them.

### NanoString nCounter analysis

RNA was analyzed by NanoString nCounter Gene Expression Analysis (NanoString Technologies) using a “CodeSet” designed in consultation with NanoString Technologies that contained probes for 72 *C. elegans* genes of interest. Probe hybridization, data acquisition and analysis were carried out per instructions from NanoString. Each RNA sample was normalized to the housekeeping genes *snb-1, ama-1, act-1, pmp-3*, and *tba-1* using the nSolver Analysis software (NanoString Technologies).

### Transmission electron microscopy

*C. elegans* were fixed overnight at 4°C in 2.5% glutaraldehyde, 1.0% paraformaldehyde in 0.05M sodium cacodylate buffer, pH 7.4 plus 3.0% sucrose. The cuticles were nicked with a razor blade in a drop of fixative under a dissecting microscope to allow the fixative to penetrate. After 1 hour fixation at room temperature, the worms were fixed overnight at 4°C. After several rinses in 0.1M cacodylate buffer, the samples were post-fixed in 1.0% osmium tetroxide in 0.1M cacodylate buffer for one hr at room temp. They were rinsed in buffer and then in double distilled water and stained, *en bloc* in 2.0% aqueous uranyl acetate for 1 hour at room temperature. After rinsing in distilled water, the last rinse was carefully drawn off and the worms were embedded in 2.0% agarose in PBS for ease of handling.

The agarose blocks were dehydrated through a graded series of ethanol to 100%, then into a 1:1 mixture of ethanol:EPON overnight on a rocker. The following day, the agarose blocks were further infiltrated in 100% EPON for several hours and then were embedded in fresh EPON overnight at 60°C. Thin sections were cut on a Reichert Ultracut E ultramicrotome, collected on formvar-coated gold grids, post-stained with uranyl acetate and lead citrate, and viewed in a JEOL 1011 TEM at 80 kV equipped with an AMT digital imaging system (Advanced Microscopy Techniques, Danvers, MA). Transmission electron microscopy studies were carried out at the MGH Microscopy Core, Program in Membrane Biology.

### Colony forming unit assay of bacterial colonization

Infected *C. elegans* were picked onto plain NGM agar plates to allow them to wriggle off any external bacteria and then picked again to another plain NGM agar plate. Afterward, worms were transferred to a 2-ml microcentrifuge tube containing 25 mM tetramisole hydrochloride and 0.01% Triton X-100 in M9W buffer to inhibit expulsion of bacteria from the worm intestine and wash any adherent external bacteria. The infected worms were washed 5–6 times in the abovementioned buffer, and the volume was brought to a total of 250 µl. 200µl of buffer was removed and plated to determine the number of colony forming units outside the worm intestine. Approximately 400 mg of 1.0-mm silicon carbide particles (Catalog no. 11079110sc; Biospec Products, Bartlesville, OK) and 100 µl of M9W buffer containing 20 mM levamisole and 2% Triton were added to each tube, and the tubes were vortexed at maximum speed for one minute to release the bacteria from the worm intestine without impairing bacterial viability. The resulting suspension was serially diluted and plated onto BHI agar plates (Nunc® OmniTray Single-Well Plates, Thermo Scientific Fisher Scientific, Waltham, MA) containing 10 µg/ml kanamycin to enumerate the number of intestinal CFUs per worm.

### Microarray analysis

RNA integrity was confirmed using a Bioanalyzer 2100 (Agilent Technologies, Santa Clara, CA) analysis. All samples had an RIN of 10. The RNA samples were hybridized to the Affymetrix *C. elegans* Genome Array GeneChips, following manufacturer protocols, at the Joslin Advanced Genomics and Genetics Core (Boston, MA).  All samples were run in triplicates, except for the heat-killed *E. coli* control, which was run in quadruplicates.

Affymetrix software was used to obtain raw probe-level chip data (.CEL files). Subsequent analysis was performed in the R programming environment following the differential gene expression analysis workflow described in [45]. Background adjustment, quantile normalization and summarization were performed on the .CEL files by Robust Multiarray Averaging (RMA) [46–48]. From this preprocessed data, differentially expressed genes were identified using the Limma (linear models for microarray data) package in R language [49]. Genes of low median intensity (< 10th percentile) were filtered and adjusted P-values were calculated using the Benjamini and Hochberg (BH) method. A log fold change > 1.0 and BH-adjusted P < 0.05 was used as a threshold to determine pathogen- or treatment-specific gene signatures. An identical analysis was performed using publicly available microarray datasets (Table S11) of *C. elegans* infection to calculate additional pathogen-specific gene signatures. Gene ontology based enrichment analysis was performed using the topGO package [50] and the results were plotted using ReViGO [51]. Microarray data are available from the National Center for Biotechnology Information/GEO repository under accession no. GSE95636.

## Supplementary Material

1430825_supp.zip

## Author summary

The enterococci are natural commensals of the human gastrointestinal tract and important hospital-borne pathogens, with the majority of human enterococcal infections caused by two species, *Enterococcus faecalis* and *Enterococcus faecium*. Many mechanistic details remain to be determined concerning how enterococci perturb their host and how the host defends itself in the wake of infection. The free-living nematode *Caenorhabditis elegans*, because of its simple body plan and amenability to genetic analysis, has been used extensively to dissect essential mechanisms of host-pathogen interactions. *E. faecalis* distends the *C. elegans* intestine, induces intestinal stasis, and later kills the worm, whereas *E. faecium* simply distends the intestine without killing. We find that these different modes of infection translate to surprisingly similar *C. elegans* gene expression profiles during infection as well as during exposure to heat-killed *E. faecalis* or *E. faecium*. On the other hand, there are also some key differences in the immune and stress pathways activated by these two enterococcal species. This study elucidates the mechanisms by which enterococci perturb host physiology, as well as the means by which *C. elegans* detects and then defends itself from these pathogens, with prospective implications for immunity and infection in all metazoans.
