# Supplementary material for: Both live and dead Enterococci activate Caenorhabditis elegans host defense via immune and stress pathways
Source: Virulence. 2018 Mar 19;9(1):683–99. doi: 10.1080/21505594.2018.1438025 (PMC5955442; doi:10.1080/21505594.2018.1438025)
Supplement: 1430825_supp.zip [file kvir-09-01-1438025-s001.zip › 1430825_supp/Supplementary_Figures_Yuen_Ausubel.pptx]

## Slide 1
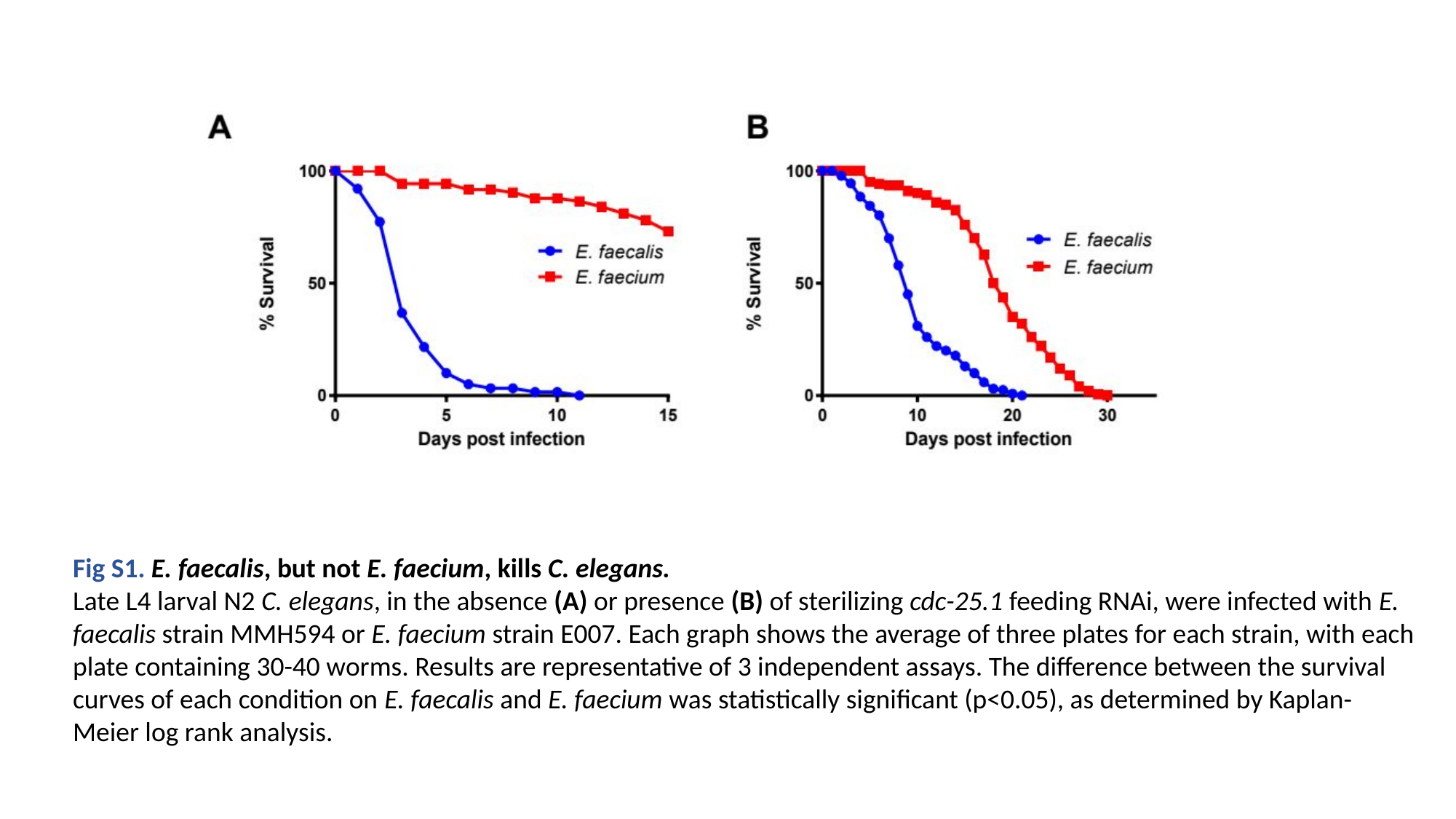

Fig S1. E. faecalis, but not E. faecium, kills C. elegans.
Late L4 larval N2 C. elegans, in the absence (A) or presence (B) of sterilizing cdc-25.1 feeding RNAi, were infected with E. faecalis strain MMH594 or E. faecium strain E007. Each graph shows the average of three plates for each strain, with each plate containing 30-40 worms. Results are representative of 3 independent assays. The difference between the survival curves of each condition on E. faecalis and E. faecium was statistically significant (p<0.05), as determined by Kaplan-Meier log rank analysis.

## Slide 2
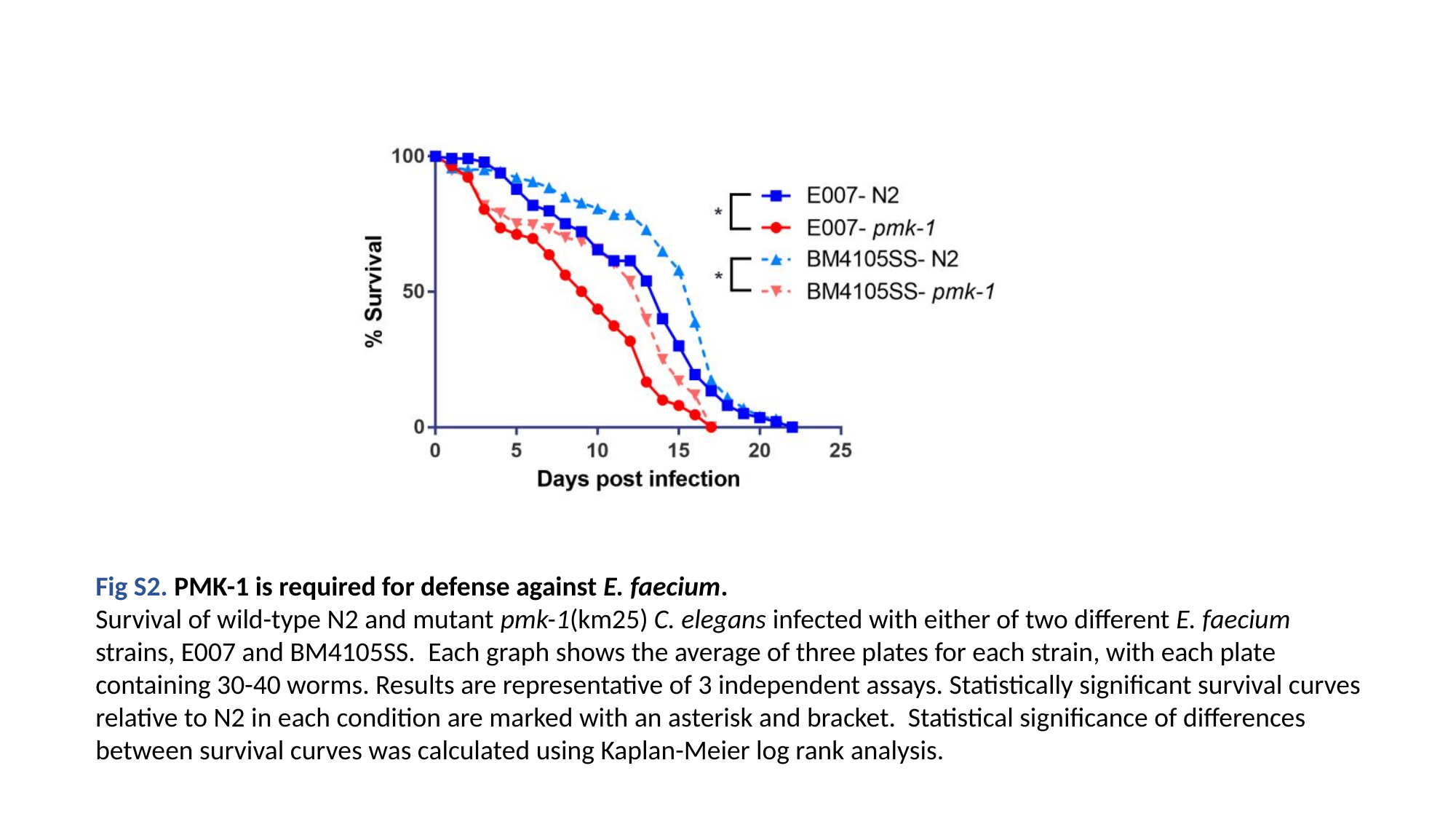

Fig S2. PMK-1 is required for defense against E. faecium.
Survival of wild-type N2 and mutant pmk-1(km25) C. elegans infected with either of two different E. faecium strains, E007 and BM4105SS. Each graph shows the average of three plates for each strain, with each plate containing 30-40 worms. Results are representative of 3 independent assays. Statistically significant survival curves relative to N2 in each condition are marked with an asterisk and bracket. Statistical significance of differences between survival curves was calculated using Kaplan-Meier log rank analysis.

## Slide 3
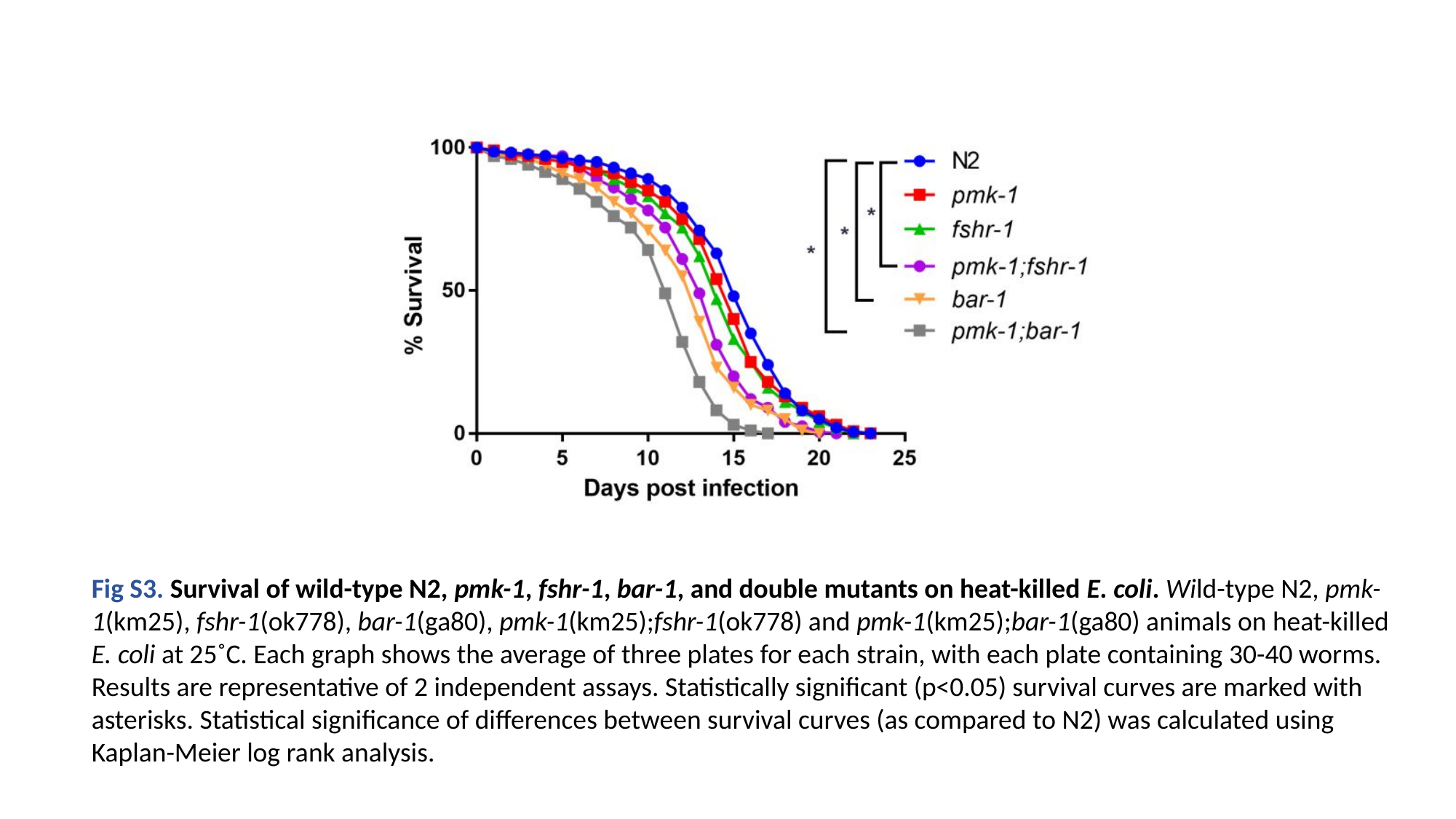

Fig S3. Survival of wild-type N2, pmk-1, fshr-1, bar-1, and double mutants on heat-killed E. coli. Wild-type N2, pmk-1(km25), fshr-1(ok778), bar-1(ga80), pmk-1(km25);fshr-1(ok778) and pmk-1(km25);bar-1(ga80) animals on heat-killed E. coli at 25˚C. Each graph shows the average of three plates for each strain, with each plate containing 30-40 worms. Results are representative of 2 independent assays. Statistically significant (p<0.05) survival curves are marked with asterisks. Statistical significance of differences between survival curves (as compared to N2) was calculated using Kaplan-Meier log rank analysis.

## Slide 4
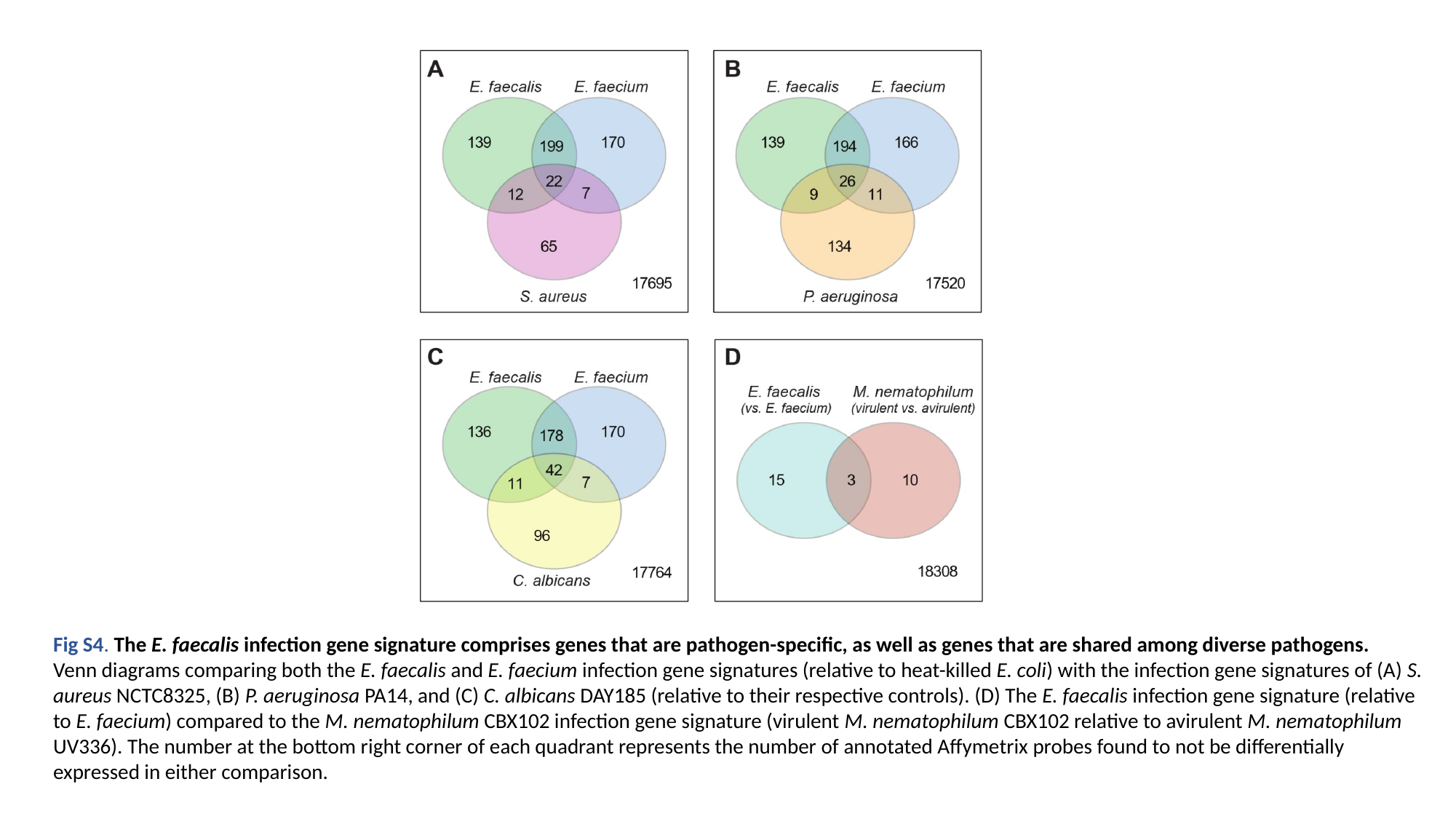

Fig S4. The E. faecalis infection gene signature comprises genes that are pathogen-specific, as well as genes that are shared among diverse pathogens.
Venn diagrams comparing both the E. faecalis and E. faecium infection gene signatures (relative to heat-killed E. coli) with the infection gene signatures of (A) S. aureus NCTC8325, (B) P. aeruginosa PA14, and (C) C. albicans DAY185 (relative to their respective controls). (D) The E. faecalis infection gene signature (relative to E. faecium) compared to the M. nematophilum CBX102 infection gene signature (virulent M. nematophilum CBX102 relative to avirulent M. nematophilum UV336). The number at the bottom right corner of each quadrant represents the number of annotated Affymetrix probes found to not be differentially expressed in either comparison.

## Slide 5
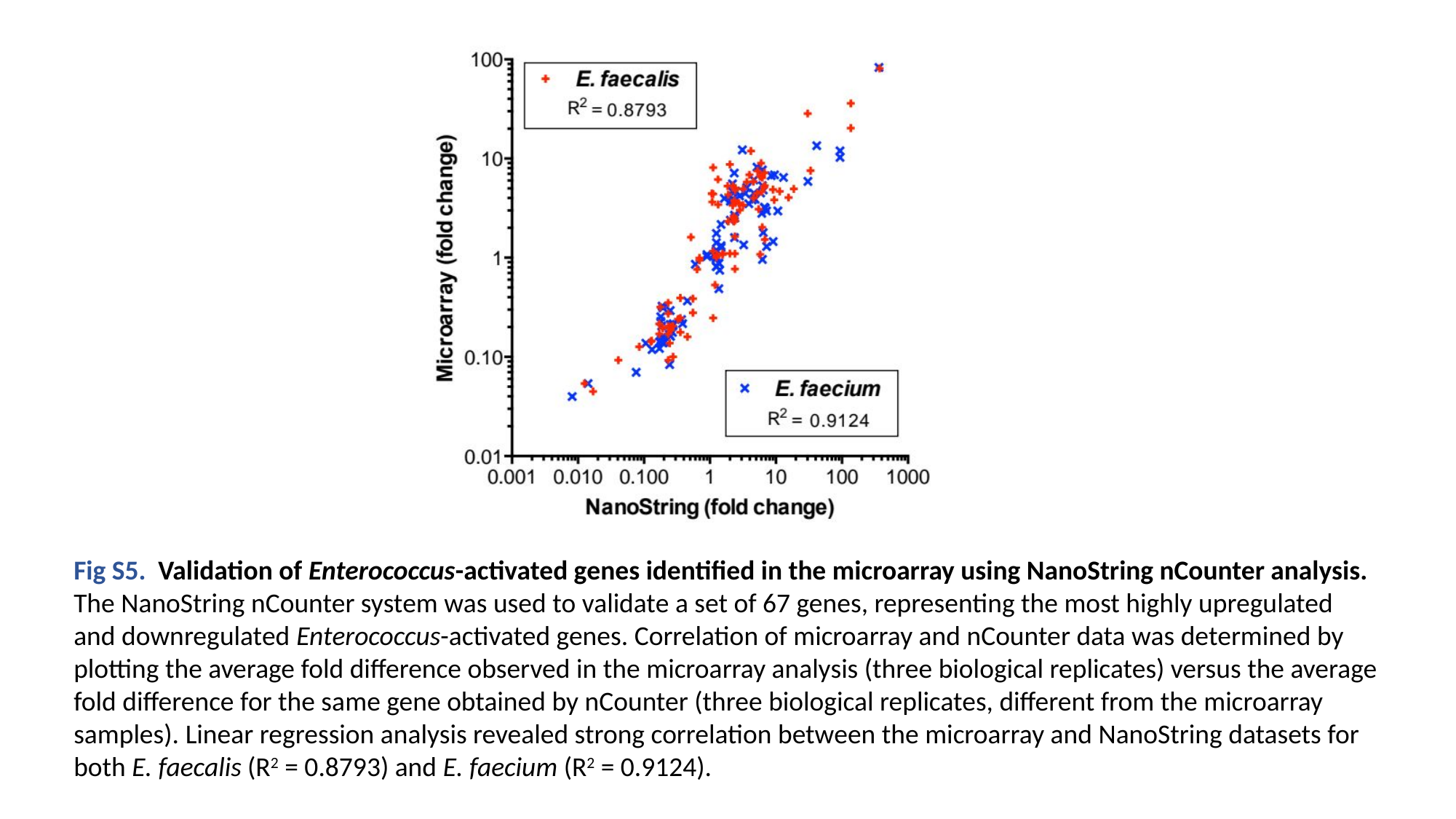

Fig S5. Validation of Enterococcus-activated genes identified in the microarray using NanoString nCounter analysis.
The NanoString nCounter system was used to validate a set of 67 genes, representing the most highly upregulated and downregulated Enterococcus-activated genes. Correlation of microarray and nCounter data was determined by plotting the average fold difference observed in the microarray analysis (three biological replicates) versus the average fold difference for the same gene obtained by nCounter (three biological replicates, different from the microarray samples). Linear regression analysis revealed strong correlation between the microarray and NanoString datasets for both E. faecalis (R2 = 0.8793) and E. faecium (R2 = 0.9124).

## Slide 6
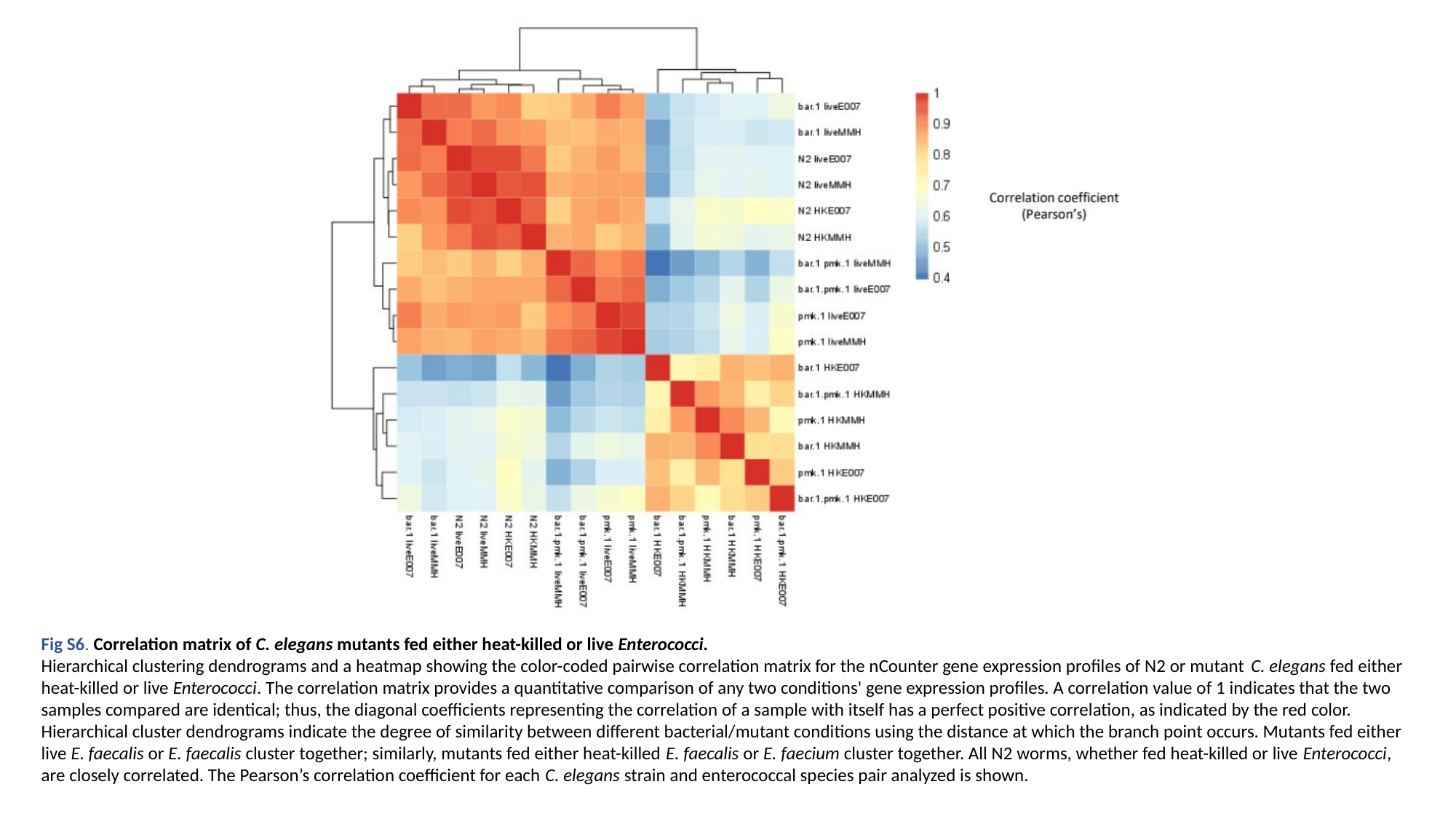

Fig S6. Correlation matrix of C. elegans mutants fed either heat-killed or live Enterococci.
Hierarchical clustering dendrograms and a heatmap showing the color-coded pairwise correlation matrix for the nCounter gene expression profiles of N2 or mutant C. elegans fed either heat-killed or live Enterococci. The correlation matrix provides a quantitative comparison of any two conditions' gene expression profiles. A correlation value of 1 indicates that the two samples compared are identical; thus, the diagonal coefficients representing the correlation of a sample with itself has a perfect positive correlation, as indicated by the red color. Hierarchical cluster dendrograms indicate the degree of similarity between different bacterial/mutant conditions using the distance at which the branch point occurs. Mutants fed either live E. faecalis or E. faecalis cluster together; similarly, mutants fed either heat-killed E. faecalis or E. faecium cluster together. All N2 worms, whether fed heat-killed or live Enterococci, are closely correlated. The Pearson’s correlation coefficient for each C. elegans strain and enterococcal species pair analyzed is shown.

## Slide 7
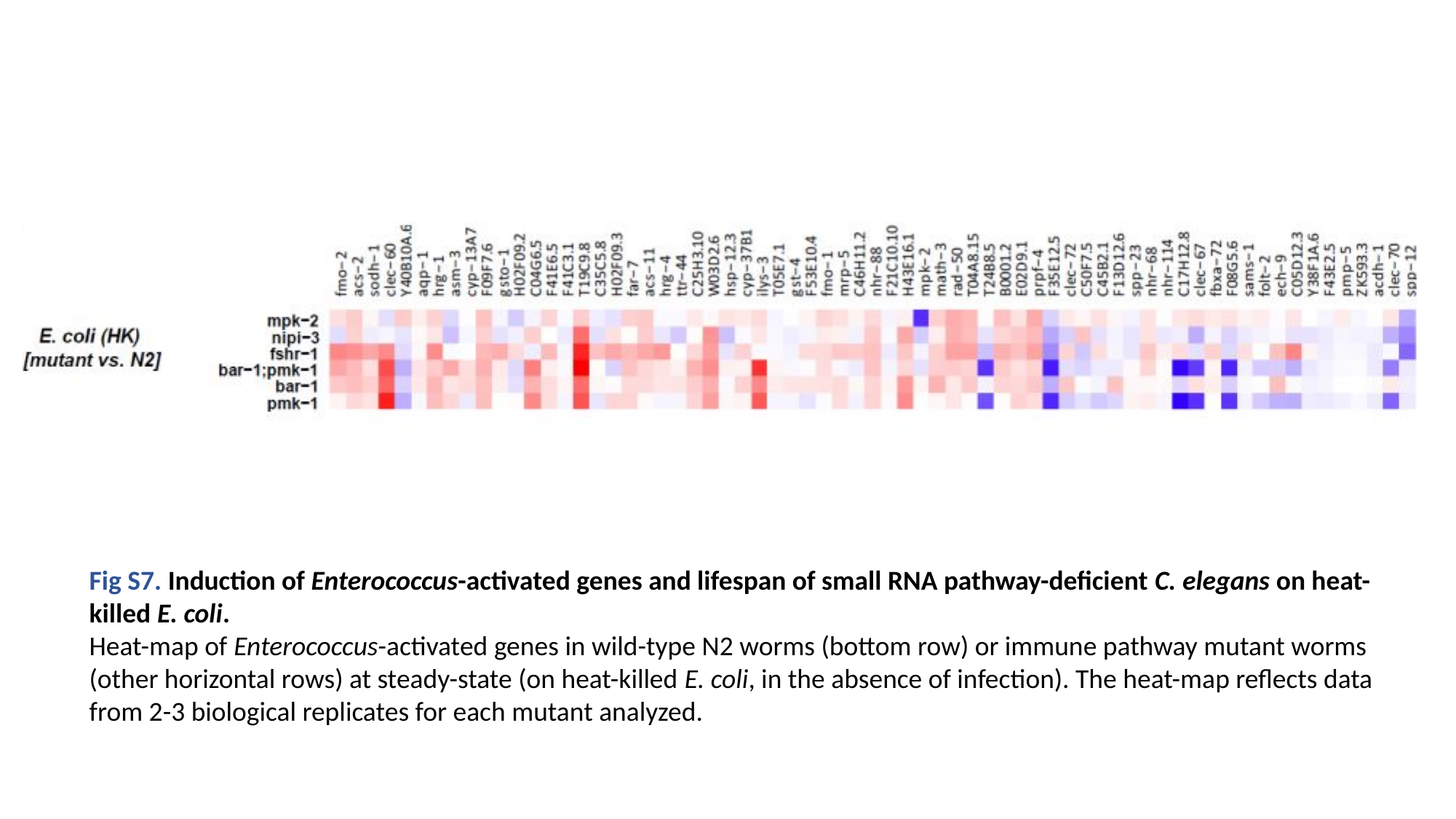

Fig S7. Induction of Enterococcus-activated genes and lifespan of small RNA pathway-deficient C. elegans on heat-killed E. coli.
Heat-map of Enterococcus-activated genes in wild-type N2 worms (bottom row) or immune pathway mutant worms (other horizontal rows) at steady-state (on heat-killed E. coli, in the absence of infection). The heat-map reflects data from 2-3 biological replicates for each mutant analyzed.

## Slide 8
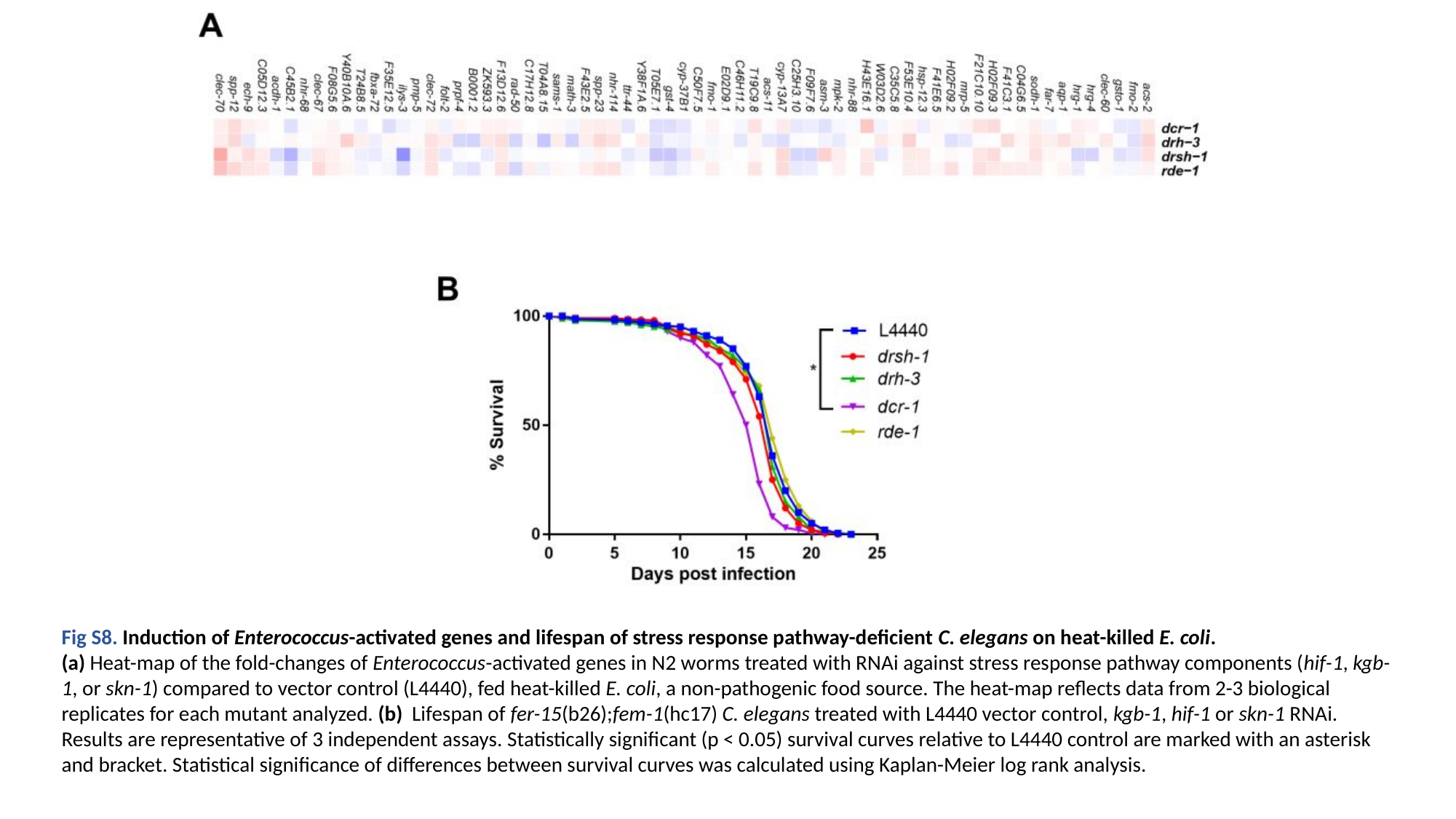

Fig S8. Induction of Enterococcus-activated genes and lifespan of stress response pathway-deficient C. elegans on heat-killed E. coli.
(a) Heat-map of the fold-changes of Enterococcus-activated genes in N2 worms treated with RNAi against stress response pathway components (hif-1, kgb-1, or skn-1) compared to vector control (L4440), fed heat-killed E. coli, a non-pathogenic food source. The heat-map reflects data from 2-3 biological replicates for each mutant analyzed. (b) Lifespan of fer-15(b26);fem-1(hc17) C. elegans treated with L4440 vector control, kgb-1, hif-1 or skn-1 RNAi. Results are representative of 3 independent assays. Statistically significant (p < 0.05) survival curves relative to L4440 control are marked with an asterisk and bracket. Statistical significance of differences between survival curves was calculated using Kaplan-Meier log rank analysis.

## Slide 9
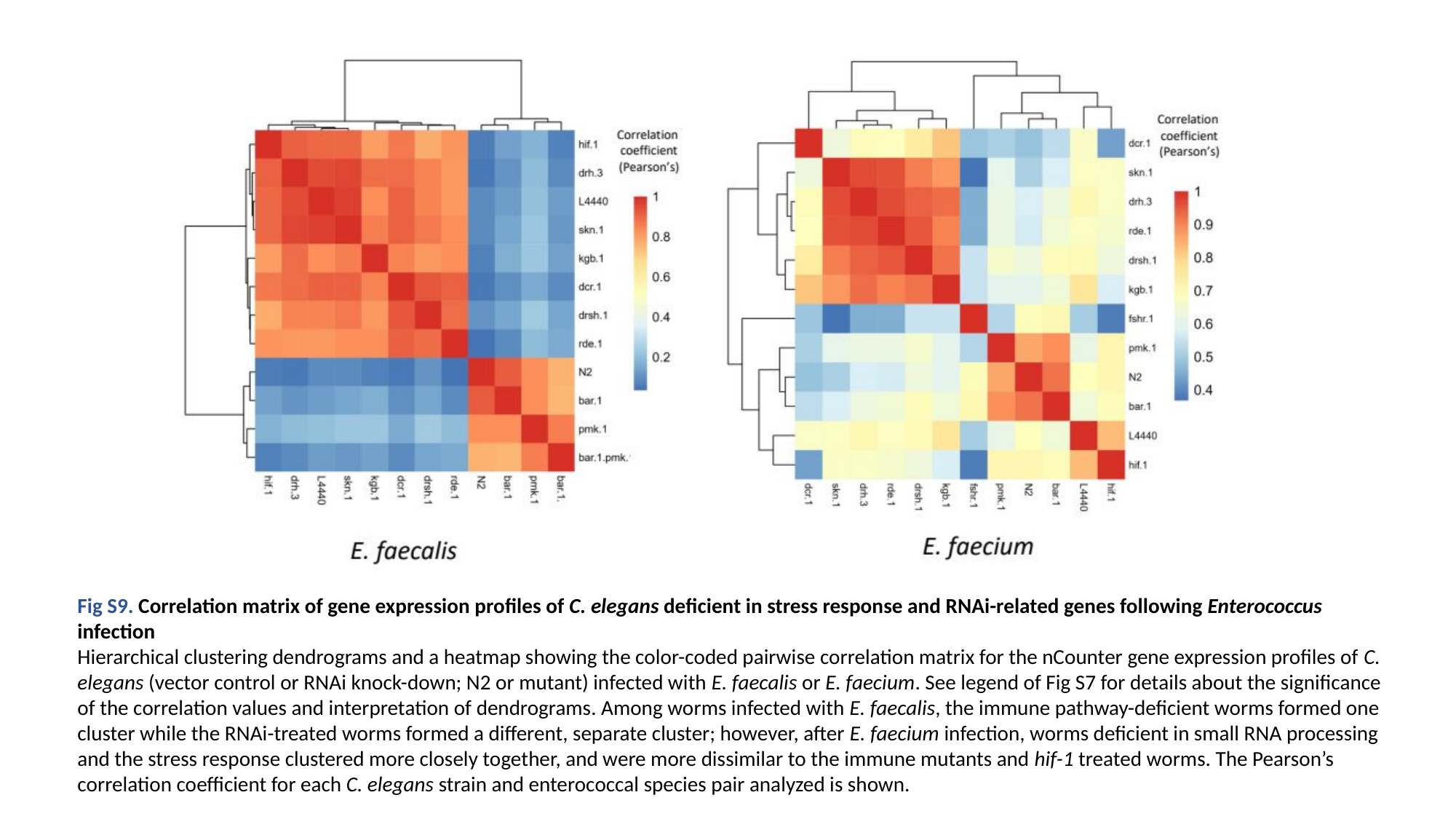

Fig S9. Correlation matrix of gene expression profiles of C. elegans deficient in stress response and RNAi-related genes following Enterococcus infection
Hierarchical clustering dendrograms and a heatmap showing the color-coded pairwise correlation matrix for the nCounter gene expression profiles of C. elegans (vector control or RNAi knock-down; N2 or mutant) infected with E. faecalis or E. faecium. See legend of Fig S7 for details about the significance of the correlation values and interpretation of dendrograms. Among worms infected with E. faecalis, the immune pathway-deficient worms formed one cluster while the RNAi-treated worms formed a different, separate cluster; however, after E. faecium infection, worms deficient in small RNA processing and the stress response clustered more closely together, and were more dissimilar to the immune mutants and hif-1 treated worms. The Pearson’s correlation coefficient for each C. elegans strain and enterococcal species pair analyzed is shown.

## Slide 10
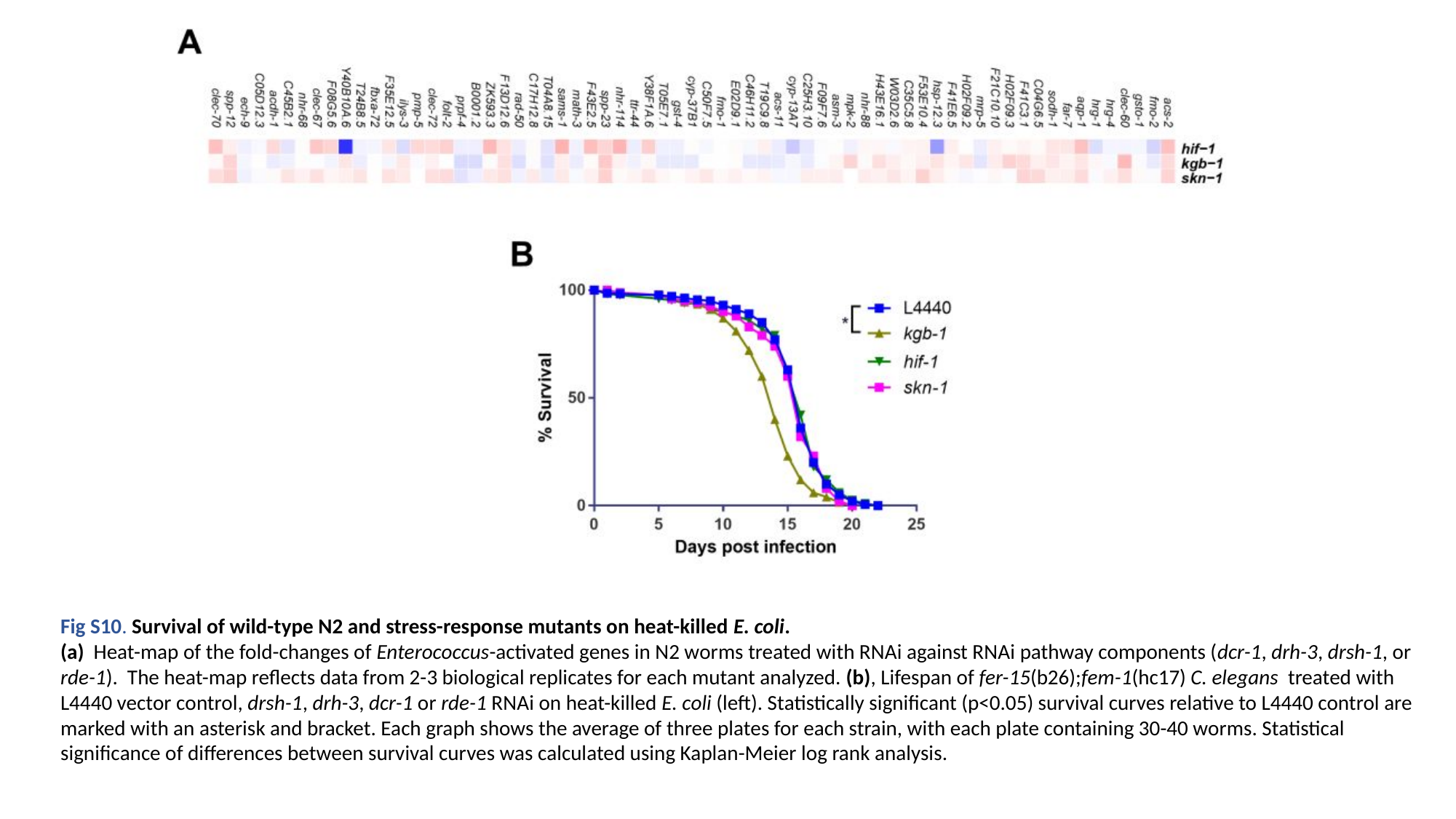

Fig S10. Survival of wild-type N2 and stress-response mutants on heat-killed E. coli.
(a) Heat-map of the fold-changes of Enterococcus-activated genes in N2 worms treated with RNAi against RNAi pathway components (dcr-1, drh-3, drsh-1, or rde-1). The heat-map reflects data from 2-3 biological replicates for each mutant analyzed. (b), Lifespan of fer-15(b26);fem-1(hc17) C. elegans treated with L4440 vector control, drsh-1, drh-3, dcr-1 or rde-1 RNAi on heat-killed E. coli (left). Statistically significant (p<0.05) survival curves relative to L4440 control are marked with an asterisk and bracket. Each graph shows the average of three plates for each strain, with each plate containing 30-40 worms. Statistical significance of differences between survival curves was calculated using Kaplan-Meier log rank analysis.

## Slide 11
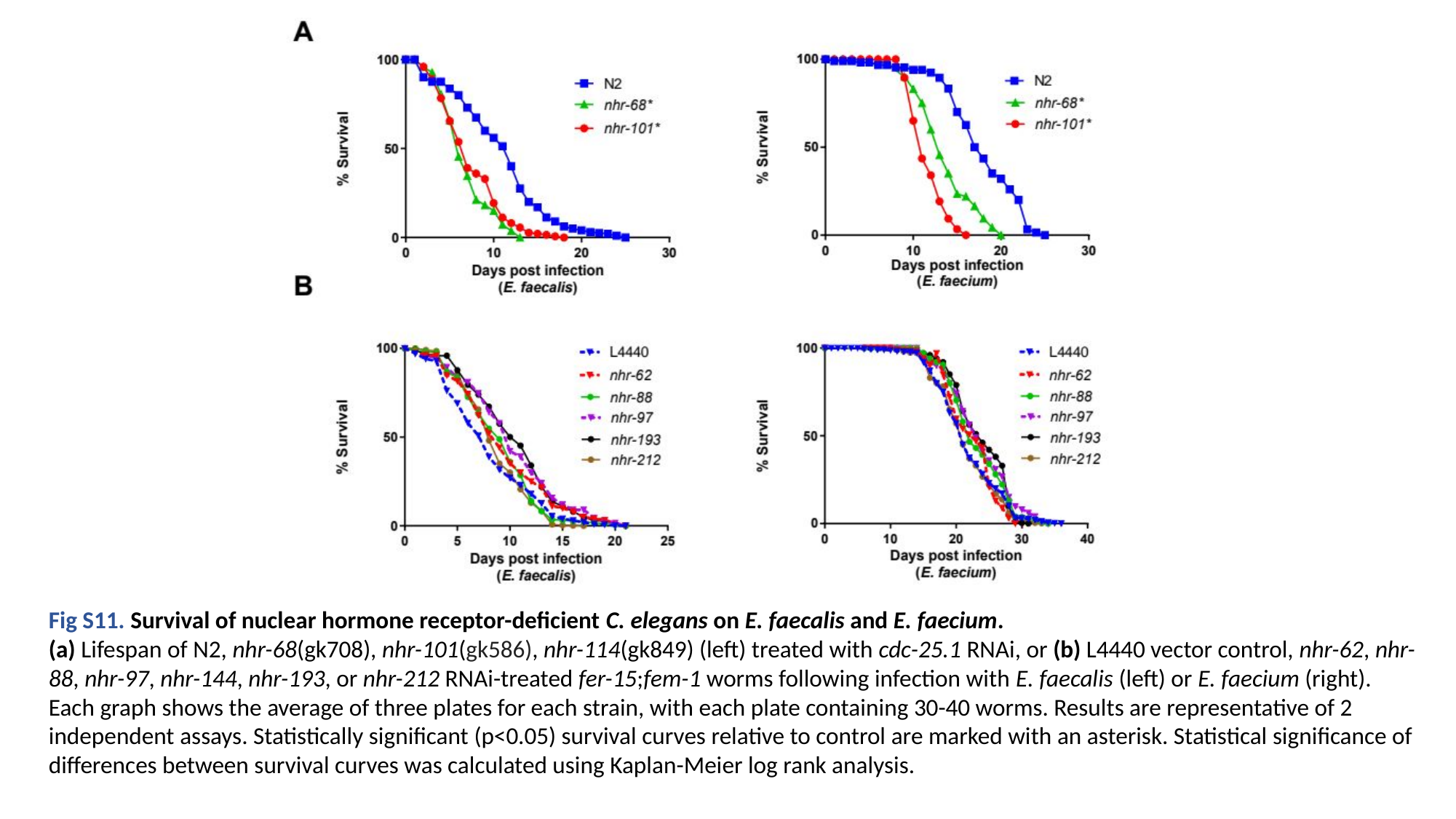

Fig S11. Survival of nuclear hormone receptor-deficient C. elegans on E. faecalis and E. faecium.
(a) Lifespan of N2, nhr-68(gk708), nhr-101(gk586), nhr-114(gk849) (left) treated with cdc-25.1 RNAi, or (b) L4440 vector control, nhr-62, nhr-88, nhr-97, nhr-144, nhr-193, or nhr-212 RNAi-treated fer-15;fem-1 worms following infection with E. faecalis (left) or E. faecium (right). Each graph shows the average of three plates for each strain, with each plate containing 30-40 worms. Results are representative of 2 independent assays. Statistically significant (p<0.05) survival curves relative to control are marked with an asterisk. Statistical significance of differences between survival curves was calculated using Kaplan-Meier log rank analysis.

## Slide 12
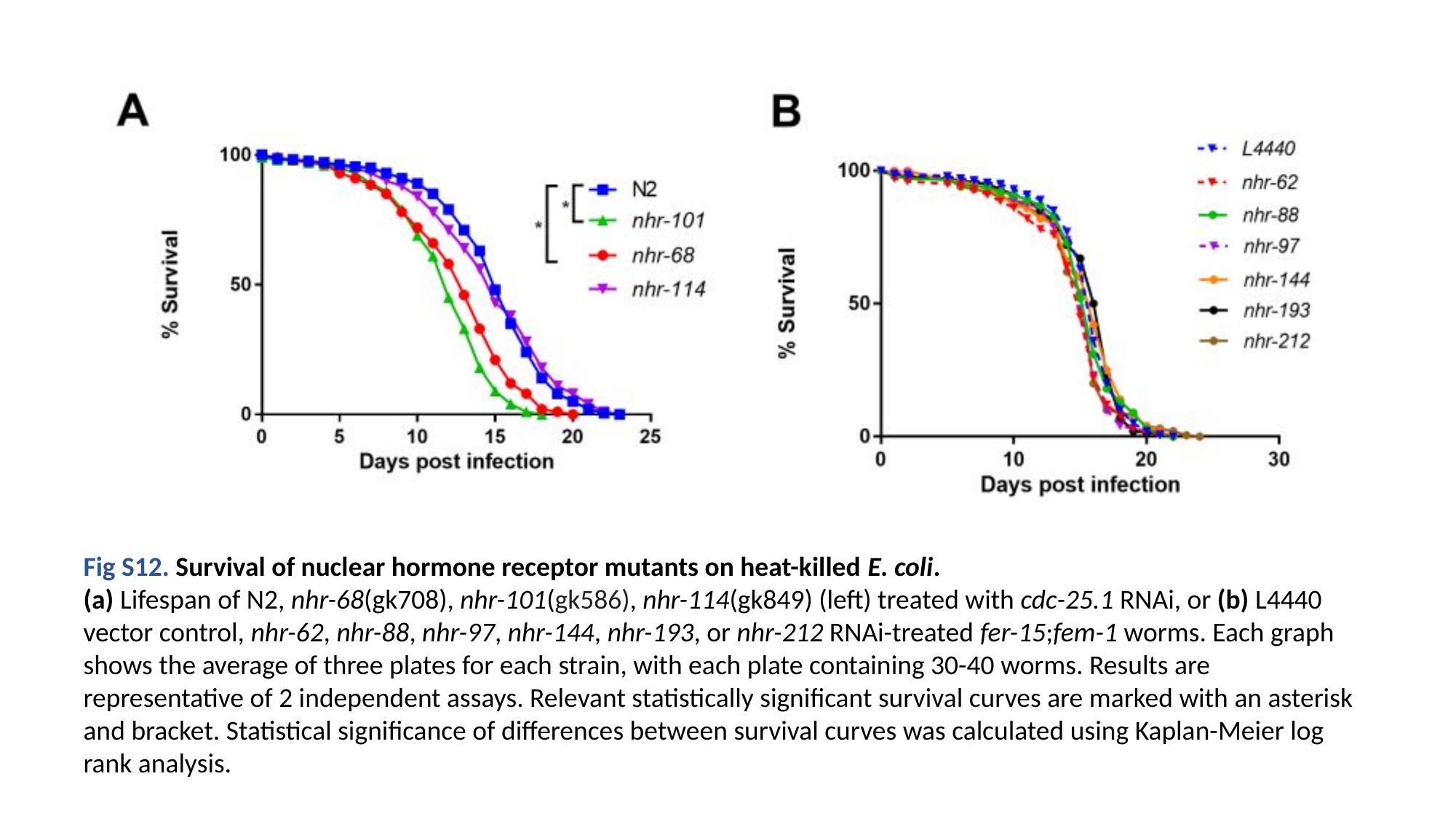

Fig S12. Survival of nuclear hormone receptor mutants on heat-killed E. coli.
(a) Lifespan of N2, nhr-68(gk708), nhr-101(gk586), nhr-114(gk849) (left) treated with cdc-25.1 RNAi, or (b) L4440 vector control, nhr-62, nhr-88, nhr-97, nhr-144, nhr-193, or nhr-212 RNAi-treated fer-15;fem-1 worms. Each graph shows the average of three plates for each strain, with each plate containing 30-40 worms. Results are representative of 2 independent assays. Relevant statistically significant survival curves are marked with an asterisk and bracket. Statistical significance of differences between survival curves was calculated using Kaplan-Meier log rank analysis.

## Slide 13
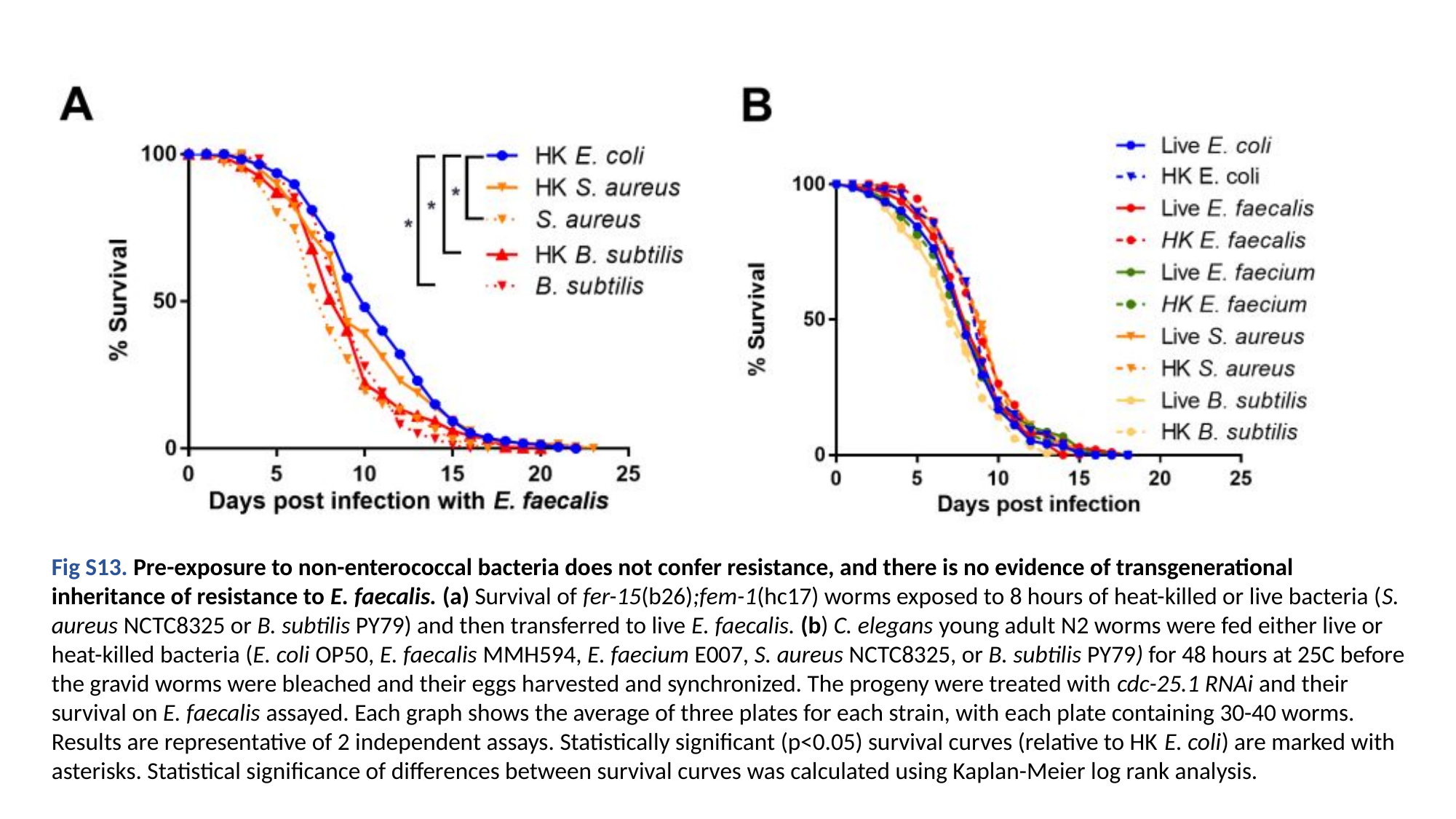

Fig S13. Pre-exposure to non-enterococcal bacteria does not confer resistance, and there is no evidence of transgenerational inheritance of resistance to E. faecalis. (a) Survival of fer-15(b26);fem-1(hc17) worms exposed to 8 hours of heat-killed or live bacteria (S. aureus NCTC8325 or B. subtilis PY79) and then transferred to live E. faecalis. (b) C. elegans young adult N2 worms were fed either live or heat-killed bacteria (E. coli OP50, E. faecalis MMH594, E. faecium E007, S. aureus NCTC8325, or B. subtilis PY79) for 48 hours at 25C before the gravid worms were bleached and their eggs harvested and synchronized. The progeny were treated with cdc-25.1 RNAi and their survival on E. faecalis assayed. Each graph shows the average of three plates for each strain, with each plate containing 30-40 worms. Results are representative of 2 independent assays. Statistically significant (p<0.05) survival curves (relative to HK E. coli) are marked with asterisks. Statistical significance of differences between survival curves was calculated using Kaplan-Meier log rank analysis.
